# Composition, Antioxidant Potential, and Antimicrobial Activity of *Helichrysum plicatum* DC. Various Extracts

**DOI:** 10.3390/plants9030337

**Published:** 2020-03-06

**Authors:** Bojan Vujić, Vera Vidaković, Milka Jadranin, Irena Novaković, Snežana Trifunović, Vele Tešević, Boris Mandić

**Affiliations:** 1 University of Belgrade-Faculty of Chemistry, Studentski trg 12-16, 11000 Belgrade, Serbia; bvujic@gmail.com (B.V.); snezanat@chem.bg.ac.rs (S.T.); vtesevic@chem.bg.ac.rs (V.T.); 2 Department of Ecology, Institute for Biological Research “Siniša Stanković”-National Institute of Republic of Serbia, University of Belgrade, Bulevar despota Stefana 142, 11060 Belgrade, Serbia; vera.vidakovic@ibiss.bg.ac.rs; 3 Institute of Chemistry, Technology and Metallurgy, National Institute, University of Belgrade, Studentski trg 12-16, 11000 Belgrade, Serbia; milkaj@chem.bg.ac.rs (M.J.); irenan@chem.bg.ac.rs (I.N.)

**Keywords:** *Helichrysum plicatum*, antioxidant potential, antimicrobial activity, solvent polarity, phenols, liquid chromatography-mass spectrometry (LC-MS)

## Abstract

*Helichrysum plicatum* DC. is widely used in folk medicine in treating a variety of health disorders. The aim of this study was to examine the influence of different extraction solvents on the chemical composition, antioxidant potential, and antimicrobial activities of *H. plicatum*. Aerial parts were separately extracted with ethanol, dichloromethane, and sunflower oil. The oil extract (OE) was re-extracted with acetonitrile. A total of 142 compounds were tentatively identified in ethanolic (EE), dichloromethane (DCME), and acetonitrile (ACNE) extracts using HPLC-DAD/ESI-ToF-MS. The dominant compound class in all extracts were α-pyrones, alongside flavonoids in EE, terpenoids in DCME and ACNE, and phloroglucinols in DCME. The antioxidant potential of the extracts was assessed by the 2,2-diphenyl-1-picrylhydrazyl radical (DPPH) assay. EE and DCME possessed the most potent radical scavenging capacity. Antimicrobial activity was investigated on eight bacterial, two yeast, and one fungal species. All extracts exhibited high antifungal and notable antibacterial activities compared to control substances, with DCME being the most potent. DCME exhibited stronger antimicrobial activity against *P. aeruginosa* than the standard chloramphenicol.

## 1. Introduction

The genus *Helichrysum* Miller (Asteraceae) comprises about 600 species of perennial or annual herbs or shrublets distributed in Europe, Asia, and Africa, the plant parts of which have been traditionally used as infusions or decoctions in the treatment of diverse ailments [1,2]. Interest in phytochemical and pharmacological studies of this genus has increased in recent years. *Helichrysum* species represent an abundant source of secondary metabolites, such as flavonoids, chalcones, phenolic acids, phthalides, coumarins, pyrones, and terpenes [1]. Plant extracts and their chemical constituents exhibit a range of biological activities, including antioxidant, anti-inflammatory, wound-healing, antimicrobial, photoprotective, anticarcinogenic, etc. [3,4,5,6,7,8,9,10,11,12].

*Helichrysum plicatum* DC. is a herbaceous perennial plant, which is native to the Balkan and Anatolian Peninsulas and Iran [13]. It has been used in folk medicine for wound-healing and the treatment of gastric and hepatic disorders, diabetes, and kidney stones [14,15,16]. Chemically, this species was mostly studied for its phenolic, mainly flavonoid content, which is thought to be responsible for the therapeutic effects of the plant. *H. plicatum* polar extracts are reported to possess antimicrobial, antidiabetic, spasmolytic, nephroprotective, and antimutagenic activities [13,17,18,19,20,21].

It is apparent that extracts from the same plant will differ in their composition of bioactive compounds depending on the extraction solvent. Since, with the exception of the essential oil, only the phenolic composition of polar extractives has been investigated in *H. plicatum* to date, the aim of this study was to perform a more detailed chemical analysis of the plant and to examine the influence of solvents of different polarity on the extraction yield, composition of extracts, antioxidant potential, and antimicrobial activities.

## 2. Results

### 2.1. Phytochemical Profile

Aerial parts of wild *H. plicatum* at the full blooming stage were extracted with solvents of different polarity. The yields were 7.14%, 2.88%, and 0.62% for ethanol (EE), dichloromethane (DCME), and acetonitrile oil (ACNE) extracts, respectively. The phytochemical content of the extracts was assessed by HPLC-DAD/ESI-ToF-MS. The peaks in chromatograms were tentatively identified on the basis of the exact molecular masses and formulas, UV spectra, and literature data (Tables S1–S3, Supplementary Material). The total ion chromatograms (TIC) in the positive (for EE and DCME) and negative (for EE, DCME and ACNE) modes and HPLC-DAD chromatograms of the investigated extracts are shown in the Supplementary Material (Figures S1–S3).

**Figure 1:**
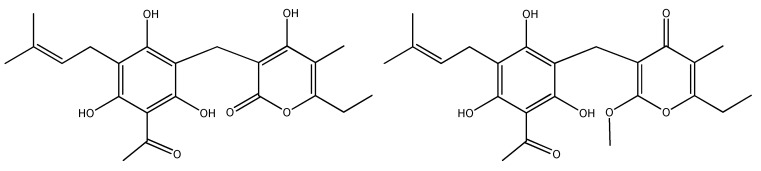
Phenolic representatives of *H. plicatum*: (**a**) naringenin (flavanone), (**b**) isosalipurposide (chalcone), (**c**) chlorogenic acid (phenolic acid ester), (**d**) 5,7-dihydroxyphthalide (phthalide), (**e**) plicatipyrone (chromanyl α-pyrone).

A total of 142 compounds were identified in *H. plicatum* aerial parts. Their distribution in the different extracts is presented in Table 1. The indicated structures are in agreement with the literature on *Helichrysum* species and/or the Asteraceae family [4,22,23,24,25,26,27,28,29,30,31,32,33,34,35,36,37,38,39,40,41,42,43,44,45,46,47,48,49,50,51,52,53,54,55,56,57,58,59,60,61,62,63,64,65,66,67,68,69,70,71,72,73,74,75,76,77,78,79,80,81,82,83,84,85,86,87,88,89,90,91,92,93,94,95,96,97,98,99,100,101,102,103,104,105,106] (Figure 1). All the extracts were characterized by α-pyrones. Simple α-pyrones (such as micropyrone and compound **1**), homodimers (helipyrone, bisnorhelipyrone), as well as heterodimers of α-pyrones and phloroglucinols (arenol, arzanol, heliarzanol, auricepyrone, etc.) were found. The prevailing compounds in EE were flavonoids. Four classes of flavonoids were identified: flavones, flavanones, flavonols, and chalcones. Compared to DCME and ACNE, only EE contained glycosylated flavonoids (compounds **43**, **44**, **58**–**60**, **63**–**65**, **67**, **68**, **70**–**72**). Being more hydrophilic than their aglycone counterparts because of the presence of sugar moieties, glycosides are easily extracted with polar protic solvents. α-Pyrones and terpenoids were predominant in both ACNE and DCME, while DCME also possessed phloroglucinols as dominant compounds (Table 1). An interesting feature of nonpolar DCM and ACN extracts is the presence of (poly)methoxylated flavones, i.e. dimethoxy- (**49**, **53**) and pentamethoxyflavones in DCME (**46**, **47**) and methoxy- (**51**, **52**), dimethoxy- (**50**), and triethoxy-dimethoxy flavones in ACNE (**54**, **55**, **57**).

### 2.2. Antioxidant Potential

The antioxidant activity of *H. plicatum* extracts was assessed by the 2,2-diphenyl-1-picrylhydrazyl (DPPH) radical assay (Table 2). EE, DCME, and ACNE demonstrated significant DPPH scavenging activity. The analysed extracts could not be compared since the polar extract (EE) was analysed by the polar DPPH method (DPPH in methanol) and nonpolar extracts (DCME, ACNE, and OE) were analysed by the nonpolar DPPH method (DPPH in toluene). However, after comparison with standards analysed by the same method, we concluded that the DCME extract exhibited a stronger antioxidant potential than the BHT standard (toluene), while EE exhibited slightly lower activity than BHT (methanol) and Trolox.

### 2.3. Antimicrobial Activity

The antimicrobial activity of *H. plicatum* was investigated against five Gram-negative bacteria (*Escherichia coli*, *Pseudomonas aeruginosa*, *Proteus hauseri*, *Klebsiella pneumoniae*, *Salmonella enterica* subsp. *enterica*), three Gram-positive bacteria (*Staphylococcus aureus*, *Bacillus subtilis*, *Clostridium sporogenes*), two yeasts (*Candida albicans*, *Saccharomyces cerevisiae*), and one fungal strain (*Aspergillus brasiliensis*) (Table 3, Table 4 and Table 5). All the extracts displayed notable antibacterial activity in the range of 0.157–2.5 mg/mL. DCME was more active than the chloramphenicol standard against *Pseudomonas aeruginosa*. In addition, DCME demonstrated the best antibacterial activity among the extracts, as indicated by the lowest minimal inhibitory concentration (MIC) values. The tested extracts exhibited better or the same antifungal activity as compared to the commercial drug nystatin. Once more, DCME had the lowest MIC values.

## 3. Discussion

### 3.1. Phytochemical Profile

The predominant compounds in EE were flavonoids and their glycosylated derivatives. Luteolin (compound **45**, Table 1), naringenin (**62**), apigenin (**48**), quercetin (**66**), kaempferol (**69**), as well as naringenin-, apigenin-, and kaempferol- glucosides (**58**–**60**, **43**, **44**, **64**) have been previously reported in *H. plicatum* [14,107]. These compounds are probably the main contributors to the therapeutic effects of the plant, which are used for treating hepatic and gastric disorders. In addition, the same flavonoids found in *H. arenarium*, which are reported to exhibit choleretic, cholagogue, hepatoprotective, and detoxifying activities, are also present in *H. plicatum* [107]. Furthermore, the phenolic-rich extracts of *H. plicatum*, with the glycosides of naringenin, apigenin, quercetin, and kaempferol as the main compounds, followed by their aglycones and chlorogenic acid, exhibited potent cytotoxic activities against human cancer cell lines [108]. Flavonoids and other phenolic constituents of water and ethanol extracts are also thought to be responsible for the antidiabetic and spasmolytic effects of this plant [17,18].

DCME and ACNE were characterized by the presence of α-pyrones. Numerous diverse pyrones were isolated from *Helichrysum* spp., although they were poorly analysed in *H. plicatum*. Plicatipyrone (**10**) was previously isolated from this species [22]. Akaberi et al. [1] listed several different groups of α-pyrone derivatives that are distributed in *Helichrysum* spp., namely monomers, glycosylated forms, homo-, and heterodimers. The α-pyrone profile tentatively determined in *H. plicatum* in the present study corresponds to α-pyrones found in other *Helichrysum* species. Vrkoč et al. [27] characterized the yellow pigment from the flowers of *H. arenarium* as a mixture of two heterodimers—phloroglucinyl α-pyrones arenol (**11**) and homoarenol (later named arzanol [109], **13**). Arzanol is probably the most investigated α-pyrone from *Helichrysum* spp. It has been reported to possess a variety of pharmacological activities, including antioxidant, anti-inflammatory, and anti-HIV [109,110,111].

Several alkoxy-flavone derivatives were detected in DCME and ACNE. *O*-alkylation increases the lipophilicity and bioavailability of these natural products. To the best of the authors’ knowledge, there are no previous records of these derivatives in *H. plicatum*, probably because this species was mostly studied for the phenolic content of more polar extracts [4,86,112,113]. Methoxylated flavones were, however, reported in other *Helichrysum* species, such as *H. viscosum* var. *bracteatum* [43], *H. decumbens* [29], *H. foetidum* [114], *H. italicum* [115], *H. kraussii* [116], *H. melaleucum* [102], *H. nitens* [42], *H. odoratissimum* [116], and *H. picardii* [115].

### 3.2. Antioxidant Potential

The results obtained in this work are in agreement with literature data indicating that the methanolic and ethanolic extracts of *H. plicatum* exhibit dose-dependent antioxidant activity. Antioxidant activity was attributed to the phenolic content of the extracts, mostly phenolic acids and flavonoids [87,108]. These compounds act by inhibiting enzymes or chelating trace elements involved in reactive oxygen species generation or by reducing highly-oxidizing free radicals through hydrogen atom donation [117,118]. The phenolic radicals generated in the process of free radical scavenging can be stabilized via intramolecular hydrogen bonds and by electron delocalization in the aromatic ring.

Comparison of the EC_50_ values with literature data obtained by different protocols and DPPH concentrations is not consistent. Kadifkova Panovska and Kulevanova [119] reported EC_50_ values in the range 6–11 mg/mL for *H. plicatum* methanol and ethanol extracts with 100 mM DPPH, while 37–88 µg/mL EC_50_ values with 0.08 mM DPPH were reported by Bigović et al. [108] and 235–918 µg/mL with 0.1 mM DPPH by Acet et al. [120]. Even though 0.1 mM DPPH was used in the present study, the results are still difficult to compare with Acet et al. because of the different mixing volumes of extracts and DPPH solutions, as well as the different incubation times. Nevertheless, compared to Trolox, the results presented herein are similar to the results from a previous study [108], whereas compared to BHT, the extracts from the present study (EE, DCME, and ACNE) were more potent in radical scavenging than the methanol and ethanol extracts of *H. plicatum* investigated by Kadifkova Panovska and Kulevanova [119].

**Figure 2:**
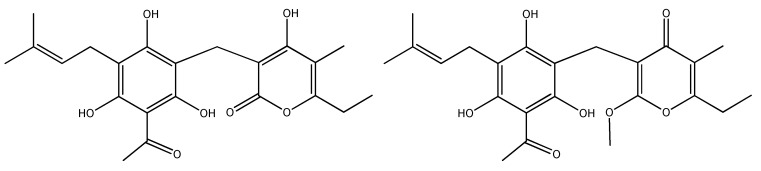
Structures of arzanol (left) and methylarzanol (right).

The most potent radical scavenging capacity in the present study was exhibited by EE and DCME, which are both rich in α-pyrones. Rosa et al. [110] showed that the α-pyrones arzanol and helipyrone achieved remarkable efficacy in scavenging lipid peroxyl radicals. Since arzanol (Figure 2) showed powerful scavenging activity against linoleic acid peroxyl radicals in the linoleic acid autoxidation test and methylarzanol was only slightly active, the authors concluded that the α-pyrone enolic hydroxyl, which can mimic a phenolic hydroxyl in assays of antioxidant activity, is responsible for the observed radical scavenging properties [110]. Furthermore, arzanol exhibited antioxidant properties at non-cytotoxic concentrations [121], which qualifies it as a candidate for investigation as a possible food additive. The presence of flavonoids probably also contributed to the significant antioxidant potential of EE.

### 3.3. Antimicrobial Activity

There are several studies on *H. plicatum* antimicrobial activity, which was ascribed to the phenolic and flavonoid contents [13,120,122]. Fungi were more sensitive to *H. plicatum* extracts than bacteria, which corroborates our findings. In previous studies, Gram-positive bacteria were more sensitive to the tested extracts than Gram-negative bacteria, although this pattern was not observed in the present study. In general, polyphenols have been widely investigated for their antimicrobial activities, which can be attributed to both direct action against microorganisms as well as the suppression of microbial virulence factors [123]. In this study, all extracts contained a variety of phenolic compounds. Furthermore, ACNE and DCME were characterized by the presence of (poly)methoxylated flavones. Methoxylated flavones from *H. nitens* displayed antifungal activity against *Cladosporium cucumerinum* [42]. This group of compounds is reported to exhibit mild antibacterial but potent fungicidal properties [124,125]. They are externally located on leaf and stem surfaces where they presumably play a role in antimicrobial defence. Methylation decreases the antioxidant potential but increases the stability of flavones, their lipophilicity, and, consequently, the ability to permeate membranes [126]. The presence of polymethoxylated flavonoids was confirmed herein in DCME, and it can be assumed that nonpolar flavonoids were responsible for the pronounced antifungal activity of this extract.

The main compounds of DCME, the most active extract in the antimicrobial tests, were α-pyrones, terpenoids, and phloroglucinols. The terpenoid components from *H. italicum*, along with flavonoids, were responsible for the antimicrobial properties of the plant [127]. Antimicrobial activity of α-pyrones from *Helichrysum* species has also been demonstrated. Four heterodimeric (italipyrone, plicatipyrone, arenol, and arzanol) and three homodimeric (helipyrone and two related compounds) α-pyrones from *H. stoechas*, which were identified in the *H. plicatum* investigated herein, displayed high activities against Gram-positive bacteria [26]. In another study, arzanol showed outstanding action against multidrug-resistant *Staphylococcus aureus* isolates, making it a good choice for further studies of suppression of antibiotic-resistant bacterial strains [23], while phloroglucinyl α-pyrones from *H. decumbens* displayed significant antifungal activity against *Cladosporium herbarum* [29].

The variety of compounds identified in *H. plicatum* most probably exert synergistic effects. Antioxidants and antimicrobials from natural sources have gained popularity over synthetic ones in the last twenty years because they most likely exhibit fewer side effects. Arenarin, which represents a mixture of phenolic antibiotics from *H. arenarium*, a plant with a similar phytochemical content to *H. plicatum*, is used as a skin and eye antibacterial agent in Russia [23]. Owing to the important biological activities of *H. plicatum* extracts and isolated compounds, they could be considered for use in the pharmaceutical, cosmetic, and food industries.

## 4. Materials and Methods

### 4.1. Plant Material

A commercial sample of wild-growing plant material was obtained from the Institute for Medicinal Plants Research “Dr. Josif Pancić”, Belgrade, Serbia. Wild-growing plant material was collected during the full-blooming stage in Macedonia in 2017.

### 4.2. Extraction Procedure

The dried and powdered plant material was extracted with 96% ethanol, dichloromethane, and sunflower oil in the dark for seven days. After filtration, solvent was removed from ethanol and dichloromethane extracts. In order to avoid ionization difficulties with the oil matrix, prior to HPLC-DAD/ESI-ToF-MS analyses, the oil extract was re-extracted with acetonitrile by overhead rotary mixer for 18 h.

### 4.3. HPLC-DAD/ESI-ToF-MS Analyses

Prior to injection, the test samples were dissolved in methanol (c ≈ 10 mg/mL) and filtered through a 0.45-μm filter.

An HPLC apparatus (Agilent 1100 Series, Agilent Technologies) with a degasser, autosampler, LiChrospher 100 RP18e column (250 × 4.0 mm i.d.; 5 μm), and a DAD detector in combination with a 6210 Time-of-Flight LC/MS system (Agilent Technologies) was used to analyse the chemical content of the tested samples. A mixture of solvents A (0.2% formic acid solution in water) and B (acetonitrile) with programmed isocratic and gradient elution was used as the mobile phase: 0–5 min 10–20% B, 5–10 min 20% B, 10–20 min 20–30% B, 20–30 min 30–70% B, 30–35 min 70–100% B, 35–40 min 100% B, 40–41 min 100–10% B, 41–45 min 10% B at a flow rate of 1.00 mL/min. The injection volume was 10 μL and the column temperature was 25°C. A DA detector was used to detect signals in the 190–550 nm wavelength range. The charged molecular ions were obtained by electrospray ionization (ESI) at atmospheric pressure: the eluted compounds were mixed with nitrogen in the heated interface and the polarity was set to negative, with the following ES parameters: capillary voltage, 4000 V; gas temperature, 350°C; drying gas flow rate, 12 L/min; nebulizer pressure, 45 psig (310.26 Pa); fragmentation voltage, 140 V, and masses were measured in the range 100–2500 *m/z*. MassHunter Workstation software was used for data recording and processing.

### 4.4. Antioxidant Assay

The antioxidant activity of the plant extracts was evaluated by the 2,2-diphenyl-1-picrylhydrazil (DPPH) assay [128], which is based on the depletion of the colour of the stable free radical DPPH in a reaction with potential antioxidants and measured by the decrease of absorbance at 517 nm. Concentrations of the extracts were in the range from 0.1 to 1.75 mg/mL. A volume of 200 µL was mixed with 1800 µL of a methanolic solution of DPPH (0.1 mM). The reaction mixture was shaken and then incubated in the dark for 30 min. After this period, the absorbance of the remaining DPPH radical was measured at 517 nm (A_sample_). Blank probes were done in the same way, using 200 µL of methanol instead of the extract solution to obtain A_blank_. All determinations were performed in triplicate. The percentage of inhibition of the DPPH radical, I(%), by each sample was calculated according to the equation:

$$I\left(\%\right)=\frac{A_{blank}-A_{sample}}{A_{blank}}\times 100.$$

The EC_50_ (concentration of an extract that reduces the absorption of DPPH solution by 50%) was calculated from the curve of the dependence of I(%) from the concentration of each extract. DPPH scavenging activity was also determined for Trolox and butylated hydroxytoluene (BHT), which are known artificial antioxidants that were used as positive probes. Tests were performed in triplicate.

The same procedure was undertaken for the determination of the radical scavenging capacity of oil extracts, except that toluene instead of methanol was used as a solvent, and BHT dissolved in toluene as a positive probe.

### 4.5. Antimicrobial Assay

Antimicrobial activity was tested against a panel of microorganisms, including: Gram-negative bacteria *Escherichia coli* (ATCC 25922), *Pseudomonas aeruginosa* (ATCC 9027), *Proteus hauseri* (ATCC 13315), *Klebsiella pneumoniae* (ATCC 10031), *Salmonella enterica* subsp. *enterica* serovar Enteritidis (ATCC 13076), Gram-positive bacteria *Staphylococcus aureus* (ATCC 6538), *Bacillus subtilis* (ATCC 6633), *Clostridium sporogenes* (ATCC 19404), yeasts *Candida albicans* (ATCC 10231), *Saccharomyces cerevisiae* (ATCC 9763), and fungal strain *Aspergillus brasiliensis* (ATCC 16404).

Antimicrobial activity was evaluated using the broth microdilution method according to the National Committee for Clinical Laboratory Standards [129]. The 96-well plates were prepared by dispensing 100 μL of Mueller-Hinton broth for bacteria and Sabouraud dextrose broth for yeasts and fungi into each well. Test extracts were dissolved in DMSO to a stock concentration of 20 mg/mL, then 100 μL from the stock solution of the tested extracts were added to the first row of the plate and double-diluted in the broth. The direct colony method was used in preparing the suspension of bacteria and yeasts in sterile 0.9% saline, while preparation of the suspension of fungal spores included gentle stripping of spores from agar slants with growing Aspergilli into sterile 0.9% saline. Suspension turbidity was conducted by comparison with 0.5 McFarland standard. After measuring the optical density OD_600_, the colony count was also checked after a series of dilutions of initial suspensions. Due to the visual detection of growth inhibition, the maximum concentrations of microorganisms were used. Ten µL of bacterial or yeast suspension or suspension of spores were added to each well to give a final concentration of 10^6^ CFU/mL for bacteria and 10^5^ CFU/mL for yeasts and fungi. In order to compare the activity of an extract with already existing, commercially available antimicrobial agents, broad-spectrum compounds commonly used in such assays served as positive controls [130]. Chloramphenicol served as the positive control for bacteria, while nystatin served as the positive control for yeasts and fungi. The inoculated plates were incubated at 37°C for 24 h for bacteria and at 28°C for 48 h for the yeasts and fungi. The MIC was determined as the lowest concentration that inhibited visible microbial growth. 

Minimum bactericidal (MBC) and minimum fungicidal concentrations (MFC) were determined by plating 10 μL of samples from wells where no colony growth was observed onto nutrient agar medium for bacteria and Sabouraud dextrose agar for yeasts and fungi. After the incubation period, the lowest concentration with no visible growth (no colony) was defined as the minimum microbicidal concentration.

## 5. Conclusions

In this study, the influence of three extraction solvents (ethanol, dichloromethane, and sunflower oil) on the composition, antioxidant, and antimicrobial activities of *Helichrysum plicatum* was investigated. The extracts comprised very diverse groups of secondary metabolites, including terpenoids and a myriad of (poly)phenolic compounds, flavonoids, α-pyrones, phloroglucinols, phenolic acids, acetophenones, phthalides, and other phenolic derivatives. EE and DCME, both rich in α-pyrones, possessed a marked antioxidant potential. DCME demonstrated the best antibacterial activity among the extracts. All extracts displayed significant antifungal capabilities, with MIC values lower than or equivalent to the MICs of the commercial antifungal agent nystatin. DCME had the lowest MIC values, which could be a consequence of the presence of nonpolar polymethoxylated flavonoids. Based on the results from this study, *H. plicatum* extracts could be considered for use as natural additives in food and cosmetic industries.

## Figures and Tables

**Table 1:** Composition of *H. plicatum* ethanol (EE), dichloromethane (DCME), and acetonitrile oil (ACNE) extract analysed by HPLC-DAD/ESI-ToF.

No	Compound Class/Name	EE	DCME	ACNE
	***Pyrone***			
	**α-pyrone**			
1	6-ethyl-4-hydroxy-5-methyl-3-(3-oxopentyl)-2H-pyran-2-one [22]			+
2	micropyrone [23]			+
3	micropyrone analog [19]			+
4	helipyrone C [20]	+	+	+
5	bisnorhelipyrone [21]		+	
6	plicatipyrone analog [22]	+		
7	3,3’-methylenebis[4-(acetyloxy)-5,6-dimethyl-2H-pyran-2-one [21]			+
8	helipyrone B [20]		+	
9	3-[[3-acetyl-2,4,6-trihydroxy-5-(3-methyl-2-buten-1-yl)phenyl]methyl]-4-hydroxy-6-methyl-2H-pyran-2-one [19]		+	
10	plicatipyrone [22]	+	+	+
11	arenol [23]	+	+	+
12	plicatipyrone analog [22]	+		
13	arzanol [27]	+	+	+
14	heliarzanol [23]	+		+
15	cycloarzanol [23]	+		
16	helipyrone diacetate [25]	+	+	
17	methylarzanol [23]	+		+
18	3-[1-[3-acetyl-2,4,6-trihydroxy-5-(3-methyl-2-buten-1-yl)phenyl]ethyl]-6-ethyl-4-hydroxy-5-methyl-2H-pyran-2-one [23]	+		+
19	auricepyrone [22], 23-methyl-6-*O*-desmethylauricepyrone [22]			+
20	helicerastripyrone [28]			+
21	4-hydroxy-5,6-dimethyl-3-[[2,4,6-trihydroxy-3-(3-methyl-2-buten-1-yl)-5-(2-methyl-1-oxobutyl)phenyl]methyl]-2H-pyran-2-one [24]	+		
22	3-[[3-acetyl-5-(3,7-dimethyl-2,6-octadien-1-yl)-2,4,6-trihydroxyphenyl]methyl]-4-hydroxy-5,6-dimethyl-2H-pyran-2-one [29]	+		
23	plicatipyrone analog [26]	+	+	
24	6-ethyl-4-hydroxy-5-methyl-3-[[2,4,6-trihydroxy-3-(3-methyl-2-buten-1-yl)-5-(2-methyl-1-oxopropyl)phenyl]methyl]-2H-pyran-2-one [23]	+		+
25	norauricepyrone [24]			+
26	3-[[2,4-dihydroxy-6-methoxy-3-(2-methyl-1-oxobutyl)phenyl]methyl]-6-ethyl-4-hydroxy-5-methyl-2H-pyran-2-one [30]			+
27	3-[1-[3-acetyl-2,4,6-trihydroxy-5-(3-methyl-2-buten-1-yl)phenyl]heptyl]-4-hydroxy-6-methyl-2H-pyran-2-one [23], 3-[[2,4-dihydroxy-6-methoxy-5-(3-methyl-2-buten-1-yl)-3-(2-methyl-1-oxobutyl)phenyl]methyl]-6-ethyl-4-hydroxy-5-methyl-2H-pyran-2-one [30], 3-[[2,4-dihydroxy-6-methoxy-3-(3-methyl-2-buten-1-yl)-5-(2-methyl-1-oxobutyl)phenyl]methyl]-6-ethyl-4-hydroxy-5-methyl-2H-pyran-2-one [22]	+		+
28	3-[[3-acetyl-5-(3,7-dimethyl-2,6-octadienyl)-2,4,6-trihydroxyphenyl]methyl]-4-hydroxy-5-methyl-6-propyl-2H-pyran-2-one [29], 3-[[3-(3,7-dimethyl-2,6-octadien-1-yl)-2,4,6-trihydroxy-5-(2-methyl-1-oxopropyl)phenyl]methyl]-4-hydroxy-5,6-dimethyl-2H-pyran-2-one [24]	+		
29	3-[[3-(3,7-dimethyl-2,6-octadien-1-yl)-2,4,6-trihydroxy-5-(2-methyl-1-oxobutyl)phenyl]methyl]-6-ethyl-4-hydroxy-5-methyl-2H-pyran-2-one [24]			+
30	3-[[3,7-dimethyl-2,6-octadien-1-yl]-2,4,6-trihydroxy-5-(2-methyl-1-oxobutyl)phenyl]methyl]-4-hydroxy-5,6-dimethyl-2H-pyran-2-one [24]	+		+
31	3-[[2,3-dihydro-4,6-dihydroxy-2-(1-methylethenyl)-5-(2-methyl-1-oxobutyl)-7-benzofuranyl]methyl]-6-ethyl-4-hydroxy-5-methyl-2H-pyran-2-one [30], 3-[[5,7-dihydroxy-2,2-dimethyl-6-(2-methyl-1-oxobutyl)-2H-1-benzopyran-8-yl]methyl]-6-ethyl-4-hydroxy-5-methyl-2H-pyran-2-one [30]			+
	**α-pyrone (coumarin)**			
32	2’,3’-dihydroxypuberulin [31]		+	
33	7-(2,3-dihydroxy-3-methylbutoxy)-5-hydroxy-6-methoxy-2H-1-benzopyran-2-one [32]	+	+	
34	coumarin derivative [33,34]		+	
35	7-(2,3-dihydroxy-3-methylbutoxy)-5,6-dimethoxy-2H-1-benzopyran-2-one [35]		+	
36	2,3-dihydro-10-methoxy-2-(1-methylethenyl)-7H-pyrano[2,3-g]-1,4-benzodioxin-7-one [36]			+
	**γ-pyrone**			
37	2-methoxy-3,5-dimethyl-6-(1-methylethyl)-4H-pyran-4-one [30]			+
38	3-[[2,4-dihydroxy-6-methoxy-3-(3-methyl-2-buten-1-yl)-5-(2-methyl-1-oxobutyl)phenyl]methyl]-6-ethyl-2-methoxy-5-methyl-4H-pyran-4-one [37]			+
39	italipyrone [22]			+
40	italipyrone analog [22]			+
	**γ-pyrone (chromone)**			
41	3-(acetyloxy)-2,3-dihydro-5,7-dihydroxy-6-(3-methyl-2-butenyl)-2-phenyl-4H-1-benzopyran-4-one [38]		+	
42	2,3-dihydro-5,7-dihydroxy-3-methyl-8-(3-methyl-2-butenyl)-2-(1-methylethyl)-4H-1-benzopyran-4-one [39]	+		
	***Flavonoid***			
	**flavone**			
43	apigenin-7-*O*-glucoside [4]	+		
44	apigenin-5-*O*-glucoside [40]	+		
45	luteolin [41]	+		
46	3,5,6,7,8-pentamethoxyflavone [42]		+	
47	3’,4’,5,6,7-pentamethoxyflavone [43]		+	
48	apigenin [40]	+		
49	dihydroxy-dimethoxyflavone [44]		+	
50	jaceosidin [45]			+
51	chrysoeriol [45]			+
52	5,7-dihydroxy-3-methoxyflavone [44]			+
53	5,7,4’-trihydroxy-3,6-dimethoxy-3’-prenylflavone [46]		+	
54	TEDMF ^1^ [43]			+
55	TEDMF [43]			+
56	6-[(6-ethyl-4-hydroxy-5-methyl-2-oxo-2H-pyran-3-yl)methyl]-2,3-dihydro-5,7-dihydroxy-8-(3-methyl-2-buten-1-yl)-2-phenyl-4H-1-benzopyran-4-one [47]		+	
57	TEDMF [43]			+
	**flavanone**			
58	naringenin-5-*O*-glucoside [40]	+		
59	naringenin-4′-*O*-glucoside [48]	+		
60	naringenin-7-*O*-glucoside [40]	+		
61	eriodictyol [49]	+		
62	naringenin [40]	+	+	
	**flavonol**			
63	quercetin-3-*O*-glucoside [4], hyperoside (quercetin-3-*O*-galactoside) [4]	+		
64	kaempferol-3-*O*-glucoside [50]	+		
65	helichrysoside [51]	+		
66	quercetin [40]	+		+
67	tiliroside [52]	+		
68	tiliroside analog [52]	+		
69	kaempferol [41]	+		
	**chalcone**			
70	arenariumoside V [53], arenariumoside VI [53], arenariumoside VII [53]	+		
71	tomoroside A [53]	+		
72	isosalipurposide [40]	+		
73	helilupolone [54]	+		
	***Terpenoid***			
	**sesquiterpenoid**			
74	sesquiterpene derivative [55,56,57]		+	
75	sesquiterpene derivative [55,56,57]		+	
76	sesquiterpene derivative [58,59,60]			+
77	sesquiterpene derivative [61,62]			+
78	sesquiterpene derivative [62,63,64]			+
79	eudesmane derivative [65]		+	
80	sesquiterpene derivative [66,67,68]			+
81	sesquiterpene derivative [66,67,68]			+
82	ainsliaside E [69]	+		
83	cinnamoyloxy-hydroxyeudesmane [70]			+
84	athrolide C [71]		+	+
85	athrolide D [71]		+	
	**diterpenoid**			
86	diterpene derivative [30,72]			+
87	8-(acetyloxy)-3-ethenyloctahydro-10-hydroxy-3,4a,7,7,10a-pentamethyl-1H-naphtho[2,1-b]pyran-2,5(3H,4aH)-dione [72]			+
88	(7-ethenyl-1,2,3,4,4a,4b,5,6,7,8,10,10a-dodecahydro-1,4a,7-trimethyl-1-phenanthrenyl)methyl-butanedioic acid methyl ester [72]			+
89	*ent*-kaurane derivative [73]		+	
90	gymnospermin [74]	+		
91	diterpene derivative [75,76,77]		+	
92	*ent*-kaurane derivative [73]		+	
93	5-(acetyloxy)-α-ethenyldecahydro-α,3a,5,7b-tetramethyl-1H-cyclopropa[a]naphthalene-4-propanol acetate [78]		+	
94	*ent*-kaurane derivative [73]	+	+	
	***Phloroglucinol***			
95	4-[3,5-dihydroxy-4-(1-oxobutyl)phenoxy]-2-methyl-butanoic acid [47], 4-[3,5-dihydroxy-4-(2-methyl-1-oxopropyl)phenoxy]-2-methyl-butanoic acid [47], 1-[2,6-dihydroxy-4-[[4-hydroxy-3-(hydroxymethyl)-2-buten-1-yl]oxy]phenyl]-1-butanone [47]		+	
96	2,3-dihydro-4,5-dimethoxy-2,2-dimethyl-6-benzofuranol [79]			+
97	4,5-dimethoxy-6-(2-methyl-1-propen-1-yl)-1,3-benzenediol-1,3-diacetate [79]		+	
98	1-(4,6-dihydroxy-2,3-dimethoxyphenyl)-2-methyl-1-butanone [43,79,80]		+	
99	4-[3,5-dihydroxy-4-(2-methyl-1-oxopropyl)phenoxy]-2-methyl-2-butenoic acid methyl ester [47]		+	
100	acylphloroglucinol derivative [47]	+	+	
101	2-methyl-1-[2,4,6-trihydroxy-3-(3-methyl-2-buten-1-yl)phenyl]-1-propanone [47], 1-[2,6-dihydroxy-4-[(3-methyl-2-buten-1-yl)oxy]phenyl]-2-methyl-1-propanone [47], 1-[2,6-dihydroxy-4-[(3-methyl-2-buten-1-yl)oxy]phenyl]-1-butanone [47], 1-[2,4,6-trihydroxy-3-(3-methyl-2-buten-1-yl)phenyl]-1-butanone [47], 1-(3,4-dihydro-5,7-dihydroxy-2,2-dimethyl-2H-1-benzopyran-6-yl)-2-methyl-1-propanone [30]		+	
102	[3,4-dihydro-5,7-dihydroxy-2-(4-methyl-3-penten-1-yl)-2H-1-benzopyran-6-yl]phenyl-methanone [81]	+	+	
103	2-(3,7-dimethyl-2,6-octadienyl)-3-hydroxy-5-methoxy-6-(2-methyl-1-oxopropyl)-2,5-cyclohexadiene-1,4-dione [30]			+
104	phenyl[2,4,6-trihydroxy-3-(3-methyl-2-buten-1-yl)phenyl]-methanone [47], [2,6-dihydroxy-4-[(3-methyl-2-buten-1-yl)oxy]phenyl]phenyl-methanone [47]	+		
105	1-[2,3-dihydro-4,6-dihydroxy-2-(1-methylethenyl)-5-benzofuranyl]-2-methyl-1-propanone [30]			+
106	7-acetyl-5’-ethyl-4,6-dihydroxy-4’-methyl-5-(3-methyl-2-buten-1-yl)-spiro[benzofuran-2(3H),2’(3’H)-furan]-3’-one [24]	+	+	
107	1-[2,6-bis(acetyloxy)-4-[[4-(acetyloxy)-3-[(acetyloxy)methyl]-2-buten-1-yl]oxy]phenyl]-1-butanone [47]	+	+	
108	4,6-dihydroxy-4’,5’-dimethyl-5-(3-methyl-2-buten-1-yl)-7-(2-methyl-1-oxobutyl)-spiro[benzofuran-2(3H),2’(3’H)-furan]-3’-one [24], 5’-ethyl-4,6-dihydroxy-4’-methyl-5-(3-methyl-2-buten-1-yl)-7-(2-methyl-1-oxopropyl)-spiro[benzofuran-2(3H),2’(3’H)-furan]-3’-one [24]			+
109	2-methyl-1-[2,4,6-trihydroxy-5-[(1S)-1-(4-hydroxy-6-methoxy-1,3-benzodioxol-5-yl)-2-methylpropyl]-3-(3-methyl-2-butenyl)phenyl]-1-propanone [30]		+	
	***Phthalide***			
110	7-(*β*-D-glucopyranosyloxy)-5-hydroxy-1(3H)-isobenzofuranone [82]	+		
111	5,7-dihydroxyphthalide [83]	+	+	
112	7-(*β*-D-glucopyranosyloxy)-5-methoxy-phthalide [83]	+		
113	5-methoxy-7-hydroxyphthalide [84]	+	+	
114	4-(4-hydroxy-3-methylbutyl)-5,7-dimethoxy-1(3H)-isobenzofuranone [85]			+
	***Phenolic acid (derivative)***			
115	chlorogenic acid [86]	+		
116	caffeic acid [87]	+		
117	everlastoside M [88]	+		
118	syringic acid [87]	+		
119	3’,4’-methylenedioxycinnamic acid [89]		+	
120	di-*O*-caffeoylquinic acid [4]	+		
121	di-*O*-caffeoylquinic acid [4]	+		
122	4-(3-methoxy-3-oxo-1-propen-1-yl)-2-(3-methyl-2-buten-1-yl)phenyl-3-(acetyloxy)-butanoic acid ester [90]	+		
	***Acetophenone***			
123	4’-hydroxy-3’-(3-methyl-2-butenyl)-acetophenone [26]			+
124	1-[2-[1-[(acetyloxy)methyl]ethenyl]-2,3-dihydro-3-hydroxy-5-benzofuranyl]ethanone [51]		+	+
	***Other***			
125	inositol [83]	+		
126	quinic acid [91]	+		
127	1,6-dihydroxy-4-oxo-2-cyclohexene-1-acetic acid methyl ester [92]		+	
128	tetrahydrojacarone [93]		+	
129	loliolide [94]		+	
130	2-[4-(1,3-dihydroxypropyl)-2-methoxyphenoxy]-3-hydroxy-1-(4-hydroxy-3-methoxyphenyl)-1-propanone [95]		+	
131	pinellic acid [96], tianshic acid [97]	+	+	
132	(3,4,5,6,7-pentamethoxy-2-benzofuranyl)phenylmethanone [98]		+	
133	PUFA ^2^ [99,100,101]	+		
134	pinoresinol [102]	+		
135	PUFA [99,100,101]		+	
136	dihydro-5-(5,8-tetradecadienyl)-2(3H)-furanone [47]		+	
137	PUFA [99,100,101]	+	+	
138	hexadecanal [103]		+	
	***Unclassified*** ^3^			
139	4-hydroxy-3,5-dimethyl-6-(1-methylethyl)-2H-pyran-2-one [30], 2-ethyl-6-methoxy-3,5-dimethyl-4H-pyran-4-one [28]			+
140	3-prenyl-2,4,6-trihydroxyacetophenone [30], 4,6-dimethoxy-5-(2-methyl-1-propen-1-yl)-1,3-benzodioxole [80]	+		+
141	helinivene A [104], piperitol [105], 1-[6-(acetyloxy)-2,3,4-trimethoxyphenyl]-3-phenyl- 2-propen-1-one [106]	+		
142	*ent*-kaurane derivative [73], eudesmane derivative [65]		+	

^1^ triethoxy-dimethoxy-flavone; ^2^ polyunsaturated fatty acid; ^3^ presumed compounds for one formula which belongs to different classes are listed as unclassified.

**Table 2:** DPPH free radical scavenging activities of *H. plicatum* ethanol (EE), dichloromethane (DCME), acetonitrile oil (ACNE), and oil extracts (OE); BHT—butylated hydroxytoluene.

	EE	DCME	ACNE	OE	Trolox	BHT (Methanol)	BHT (Toluene)
EC_50_^1^ (mg/mL)	0.45 ± 0.04	0.58 ± 0.02	1.74 ± 0.01	17.61 ± 0.16	0.064 ± 0.01	0.33 ± 0.01	1.42 ± 0.01

^1^ Data are presented as mean ± standard deviation (*n* = 3).

**Table 3:** Antibacterial activity of *H. plicatum* ethanol (EE), dichloromethane (DCME), and acetonitrile oil (ACNE) extract against Gram-negative bacteria.

	*Escherichia coli*		*Pseudomonas aeruginosa*		*Proteus hauseri*		*Klebsiella pneumoniae*		*Salmonella enterica* subsp. *enterica*	
	MIC	MBC	MIC	MBC	MIC	MBC	MIC	MBC	MIC	MBC
	mg/mL		mg/mL		mg/mL		mg/mL		mg/mL	
EE	2.5	>10	0.625	2.5	2.5	>10	1.25	5	2.5	5
DCME	0.313	2.5	**0.157** ^1^	0.625	0.157	0.625	0.157	0.625	1.25	10
ACNE	0.625	2.5	1.25	5	1.25	5	0.625	1.25	1.25	5
Chloramphenicol	0.062		0.25		0.125		0.062		0.125	

^1^ MIC value lower than the control.

**Table 4:** Antibacterial activity of *H. plicatum* ethanol (EE), dichloromethane (DCME), and acetonitrile oil (ACNE) extract against Gram-positive bacteria.

	*Staphylococcus aureus*		*Bacillus subtilis*		*Clostridium sporogenes*	
	MIC	MBC	MIC	MBC	MIC	MBC
	mg/mL		mg/mL		mg/mL	
EE	0.313	2.5	0.313	1.25	2.5	10
DCME	0.157	0.625	0.157	0.625	0.313	1.25
ACNE	0.625	5	2.5	10	2.5	10
Chloramphenicol	0.015		0.015		0.25	

**Table 5:** Antifungal activity of *H. plicatum* ethanol (EE), dichloromethane (DCME), and acetonitrile oil (ACNE) extract.

	*Aspergillus brasiliensis*		*Saccharomyces cerevisiae*		*Candida albicans*	
	MIC	MFC	MIC	MFC	MIC	MFC
	mg/mL		mg/mL		mg/mL	
EE	**1.25** ^1^	>10	1.25	10	2.5	5
DCME	**0.625**	2.5	1.25	5	**1.25**	2.5
ACNE	**1.25**	10	1.25	10	2.5	5
Nystatin	2.5		1.25		2.5	

^1^ MIC values lower than the control are bolded.

## Supplementary Materials

The following are available online at https://www.mdpi.com/2223-7747/9/3/337/s1, Figure S1: The total ion and HPLC-DAD chromatograms of *Helichrysum plicatum* ethanol extract (EE), Figure S2: The total ion and HPLC-DAD chromatograms of *Helichrysum plicatum* dichloromethane extract (DCME), Figure S3: The total ion and HPLC-DAD chromatograms of *Helichrysum plicatum* acetonitrile oil extract (ACNE), Table S1: Tentative analysis of *Helichrysum plicatum* ethanol extract (EE), Table S2: Tentative analysis of *Helichrysum plicatum* dichloromethane extract (DCME), Table S3: Tentative analysis of *Helichrysum plicatum* acetonitrile oil extract (ACNE).
