# Supplementary material for: Composition, Antioxidant Potential, and Antimicrobial Activity of Helichrysum plicatum DC. Various Extracts
Source: Plants (Basel). 2020 Mar 6;9(3):337. doi: 10.3390/plants9030337 (PMC7154845; doi:10.3390/plants9030337)
Supplement: Supplementary file 1 [file plants-09-00337-s001.zip › Table S3_ACNE tentative.docx]

**Table S3.** Tentative analysis of *Helichrysum plicatum* acetonitrile oil extract (ACNE).

| t_R_ (min) | | UV λ_max_ (nm) | ToF-MS (*m/z*) | formula | compound |
| --- | --- | --- | --- | --- | --- |
| ESI^-^ | LC-DAD |  | [ESI^-^] |  |  |
| 2.32 |  |  | 191.0180 [M-H]^-^ | C_6_H_8_O_7_ | n.i. |
| 10.25 |  |  | 233.0818 [M-H]^-^ | C_13_H_14_O_4_ | gnaphaliol [51] |
|  |  |  | 467.1705 [2M-H]^-^ |  |  |
| 10.25 |  |  | 209.0456 [M-H]^-^ | C_10_H_10_O_5_ | 2,4-diacetylphloroglucinol [107] |
| 15.36 |  |  | 223.0971 [M-H]^-^ | C_12_H_16_O_4_ | 2,​3-​dihydro-​4,​5-​dimethoxy-​2,​2-​dimethyl-6-​benzofuranol [79] |
| 19.28 |  |  | 181.0861 [M-H]^-^ | C_10_H_14_O_3_ | 4-​hydroxy-​3,​5-​dimethyl-​6-​(1-​methylethyl)​-2H-​pyran-​2-​one [30], 2-ethyl-6-methoxy-3,5-dimethyl-4H-pyran-4-one [28] |
| 20.94 | 20.70 | 290 | 237.1124 [M-H]^-^ | C_13_H_18_O_4_ | 6-​ethyl-​4-​hydroxy-​5-​methyl-​3-​(3-​oxopentyl)​-2H-​pyran-​2-​one [22] |
| 21.68 | 21.44 | 200; 224; 276 | 321.0971 [M+HCO_2_]^-^ | C_15_H_16_O_5_ | n.i. |
| 22.90 | 22.66 | 256; 370 | 301.0348 [M-H]^-^ | C_15_H_10_O_7_ | quercetin [40] |
| 24.66 | 24.37 | 294 | 195.1022 [M-H]^-^ | C_11_H_16_O_3_ | 2-methoxy-3,5-dimethyl-6-(1-methylethyl)-4H-pyran-4-one [30] |
| 25.06 | 24.75 | 240sh; 290 | 251.1281 [M-H]^-^ | C_14_H_20_O_4_ | micropyrone [23] |
| 25.64 | 25.35 | 290 | 251.1272 [M-H]^-^ | C_14_H_20_O_4_ | micropyrone analog [23] |
| 26.72 |  |  | 329.0657 [M-H]^-^ | C_17_H_14_O_7_ | jaceosidin [45] |
| 27.29 | 26.97 | 238sh; 278 | 299.0555 [M-H]^-^ | C_16_H_12_O_6_ | chrysoeriol [45] |
| 27.76 |  |  | 293.1020 [M-H]^-^ | C_15_H_18_O_6_ | helipyrone C [24] |
| 28.22 | 27.90 | 226; 282 | 279.1227 [M-H]^-^ | C_15_H_20_O_5_ | 4-​(4-​hydroxy-​3-​methylbutyl)​-​5,​7-​dimethoxy-1(3H)​-​isobenzofuranone [85] |
| 28.65 |  |  | 359.1854 [M-H]^-^ | C_21_H_28_O_5_ | 2-​(3,​7-​dimethyl-​2,​6-​octadienyl)​-​3-​hydroxy-​5-​methoxy-​6-​(2-​methyl-​1-​oxopropyl)​-2,​5-​cyclohexadiene-​1,​4-​dione [30] |
| 28.96 | 28.63 | 230; 282 | 203.1071 [M-H]^-^ | C_13_H_16_O_2_ | 4'-hydroxy-3'-(3-methyl-2-butenyl)-acetophenone [26] |
| 29.52 | 29.19 | 228sh; 290 | 235.0962 [M-H]^-^ | C_13_H_16_O_4_ | 3-prenyl-2,4,6-trihydroxyacetophenone [30], 4,​6-​dimethoxy-​5-​(2-​methyl-​1-​propen-​1-​yl)​-1,​3-​benzodioxole [80] |
| 29.77 |  |  | 375.1075 [M-H]^-^ | C_19_H_20_O_8_ | 3,​3'-​methylenebis[4-​(acetyloxy)​-​5,​6-​dimethyl-2H-​pyran-​2-​one [25] |
| 30.04 | 29.69 | 226; 280 | 319.0817 [M+HCO_2_]^-^ | C_15_H_14_O_5_ | 2,3-dihydro-10-methoxy-2-(1-methylethenyl)-7H-pyrano[2,3-g]-1,4-benzodioxin-7-one [36] |
| 30.10 |  |  | 459.1648 [M-H]^-^ | C_24_H_28_O_9_ | n.i. |
| 30.33 | 29.99 | 266; 348sh | 283.0604 [M-H]^-^ | C_16_H_12_O_5_ | 5,7-dihydroxy-3-methoxyflavone [44] |
| 30.60 |  |  | 321.1360 [M-H]^-^ | C_17_H_22_O_6_ | sesquiterpene derivative [58–60] |
| 30.79 |  |  | 387.1437 [M-H]^-^ | C_21_H_24_O_7_ | arenol [27] |
| 30.97 |  |  | 417.1544 [M-H]^-^ | C_22_H_26_O_8_ | plicatipyrone [26] |
| 31.10 |  |  | 387.2531 [M-H]^-^ | C_24_H_36_O_4_ | diterpene derivative [30,72] |
|  |  |  | 775.5163 [2M-H]^-^ |  |  |
| 31.24 |  |  | 391.2089 [M-H]^-^ | C_22_H_32_O_6_ | 8-​(acetyloxy)​-​3-​ethenyloctahydro-​10-​hydroxy-​3,4a,​7,​7,​10a-​pentamethyl-​1H-​naphtho[2,​1-​b]​pyran-​2,5(3H,4aH)-​dione [72] |
| 31.37 |  |  | 333.0978 [M-H]^-^ | C_17_H_18_O_7_ | sesquiterpene derivative [61,62] |
| 31.45 |  |  | 275.0919 [M-H]^-^ | C_15_H_16_O_5_ | 1-[2-[1-[(acetyloxy)methyl]ethenyl]-2,3-dihydro-3-hydroxy-5-benzofuranyl]ethanone [51] |
| 31.75 | 31.41 | 294 | 401.1616 [M-H]^-^ | C_22_H_26_O_7_ | arzanol [27] |
| 31.94 | 31.61 | 242sh; 292 | 445.1847 [M-H]^-^ | C_24_H_30_O_8_ | heliarzanol [23] |
| 32.27 |  |  | 261.1125 [M-H]^-^ | C_15_H_18_O_4_ | 1-​[2,​3-​dihydro-​4,​6-​dihydroxy-​2-​(1-​methylethenyl)​-​5-​benzofuranyl]​-​2-​methyl-1-​propanone [30] |
|  |  |  | 307.1180 [M+HCO_2_]^-^ |  |  |
| 32.56 | 32.20 | 236; 292 | 415.1738 [M-H]^-^ | C_23_H_28_O_7_ | methylarzanol [23] |
| 33.07 | 32.71 | 292 | 415.1754 [M-H]^-^ | C_23_H_28_O_7_ | 3-​[1-​[3-​acetyl-​2,​4,​6-​trihydroxy-​5-​(3-​methyl-​2-​buten-​1-​yl)​phenyl]​ethyl]​-​6-​ethyl-​4-​hydroxy-​5-​methyl-2H-pyran-​2-​one [23] |
| 33.26 | 32.92 | 208; 292 | 315.1981 [M-H]^-^ | C_20_H_28_O_3_ | n.i. |
| 33.70 | 33.32 | 294 | 429.1898 [M-H]^-^ | C_24_H_30_O_7_ | 6-​ethyl-​4-​hydroxy-​5-​methyl-​3-​[[2,​4,​6-​trihydroxy-​3-​(3-​methyl-​2-​buten-​1-​yl)​-​5-​(2-​methyl-​1-​oxopropyl)​phenyl]​methyl]​-2H-​pyran-​2-​one [23] |
| 33.98 |  |  | 361.1284 [M-H]^-^ | C_19_H_22_O_7_ | sesquiterpene derivative [62–64] |
| 34.32 | 33.94 | 234sh; 296 | 443.2047 [M-H]^-^ | C_25_H_32_O_7_ | auricepyrone [22], 23-methyl-6-*O*-desmethylauricepyrone [22] |
| 34.44 | 34.05 | 236sh; 296 | 469.2208 [M-H]^-^ | C_27_H_34_O_7_ | helicerastripyrone [28] |
| 34.76 | 34.37 | 240sh; 292 | 375.1441 [M-H]^-^ | C_20_H_24_O_7_ | norauricepyrone [24] |
| 34.90 |  |  | 457.2213 [M-H]^-^ | C_26_H_34_O_7_ | 3-​[1-​[3-​acetyl-​2,​4,​6-​trihydroxy-​5-​(3-​methyl-​2-​buten-​1-​yl)​phenyl]​heptyl]​-​4-​hydroxy-​6-​methyl-2H-​pyran-​2-​one [23], 3-​[[2,​4-​dihydroxy-​6-​methoxy-​5-​(3-​methyl-​2-​buten-​1-​yl)​-​3-​(2-​methyl-​1-​oxobutyl)​phenyl]​methyl]​-​6-​ethyl-​4-​hydroxy-​5-​methyl-2H-​pyran-​2-​one [30], 3-​[[2,​4-​dihydroxy-​6-​methoxy-​3-​(3-​methyl-​2-​buten-​1-​yl)​-​5-​(2-​methyl-​1-​oxobutyl)​phenyl]​methyl]​-​6-​ethyl-​4-​hydroxy-​5-​methyl-2H-​pyran-​2-​one [22] |
| 35.25 |  |  | 431.1700 [M-H]^-^ | C_23_H_28_O_8_ | sesquiterpene derivative [66–68] |
| 35.34 |  |  | 471.2373 [M-H]^-^ | C_27_H_36_O_7_ | 3-[[2,4-dihydroxy-6-methoxy-3-(3-methyl-2-buten-1-yl)-5-(2-methyl-1-oxobutyl)phenyl]methyl]-6-ethyl-2-methoxy-5-methyl-4H-pyran-4-one [37] |
| 35.52 |  |  | 389.1594 [M-H]^-^ | C_21_H_26_O_7_ | 3-​[[2,​4-​dihydroxy-​6-​methoxy-​3-​(2-​methyl-​1-​oxobutyl)​phenyl]​methyl]​-​6-​ethyl-​4-​hydroxy-​5-​methyl-2H-​pyran-​2-​one [30] |
| 35.56 |  |  | 413.1596 [M-H]^-^ | C_23_H_26_O_7_ | TEDMF^1^ [43] |
| 35.68 |  |  | 431.1702 [M-H]^-^ | C_23_H_28_O_8_ | sesquiterpene derivative [66–68] |
| 35.75 |  |  | 497.2532 [M-H]^-^ | C_29_H_38_O_7_ | 3-​[[3,​7-​dimethyl-​2,​6-​octadien-​1-​yl]​-​2,​4,​6-​trihydroxy-​5-​(2-​methyl-​1-​oxobutyl)​phenyl]​methyl]​-​4-​hydroxy-​5,​6-​dimethyl-2H-pyran-​2-​one [24] |
| 35.96 | 35.57 | 210; 292 | 413.1592 [M-H]^-^ | C_23_H_26_O_7_ | TEDMF [43] |
|  |  |  | 873.3323 [2M+HCO_2_]^-^ |  |  |
| 36.01 |  |  | 369.2425 [M-H]^-^ | C_24_H_34_O_3_ | cinnamoyloxy-hydroxyeudesmane [70] |
| 36.12 |  |  | 511.2686 [M-H]^-^ | C_30_H_40_O_7_ | 3-​[[3-​(3,​7-​dimethyl-​2,​6-​octadien-​1-​yl)​-​2,​4,​6-​trihydroxy-​5-​(2-​methyl-​1-​oxobutyl)​phenyl]​methyl]​-​6-​ethyl-​4-​hydroxy-​5-​methyl-2H-​pyran-​2-​one [24] |
| 36.34 |  |  | 399.1797 [M-H]^-^ | C_23_H_28_O_6_ | 4,​6-​dihydroxy-​4',​5'-​dimethyl-​5-​(3-​methyl-​2-​buten-​1-​yl)​-​7-​(2-​methyl-​1-​oxobutyl)​-spiro[benzofuran-​2(3H)​,​2'(3'H)​-​furan]​-​3'-​one [24], 5'-​ethyl-​4,​6-​dihydroxy-​4'-​methyl-​5-​(3-​methyl-​2-​buten-​1-​yl)​-​7-​(2-​methyl-​1-​oxopropyl)​-spiro[benzofuran-​2(3H)​,​2'(3'H)​-​furan]​-​3'-​one [24] |
|  |  |  | 445.1852 [M+HCO_2_]^-^ |  |  |
| 36.51 |  |  | 427.1751 [M-H]^-^ | C_31_H_42_O_7_ | n.i. |
| 36.54 |  |  | 399.1439 [M-H]^-^ | C_22_H_24_O_7_ | italipyrone [22] |
| 36.62 |  |  | 483.2015 [M-H]^-^ | C_27_H_32_O_8_ | n.i. |
|  |  |  | 967.4067 [2M-H]^-^ |  |  |
| 36.82 |  |  | 401.2686 [M-H]^-^ | C_25_H_38_O_4_ | (7-​ethenyl-​1,​2,​3,​4,​4a,​4b,​5,​6,​7,​8,​10,​10a-​dodecahydro-​1,​4a,​7-​trimethyl-​1-​phenanthrenyl)​methyl-butanedioic acid methyl ester [72] |
|  |  |  | 447.2752 [M+HCO_2_]^-^ |  |  |
| 36.87 |  |  | 459.2007 [M-H]^-^ | C_25_H_32_O_8_ | athrolide C [71] |
| 37.10 |  |  | 441.1902 [M-H]^-^ | C_25_H_30_O_7_ | 3-​[[2,​3-​dihydro-​4,​6-​dihydroxy-​2-​(1-​methylethenyl)​-​5-​(2-​methyl-​1-​oxobutyl)​-​7-​benzofuranyl]​methyl]​-​6-​ethyl-​4-​hydroxy-​5-​methyl-2H-pyran-​2-​one [30], 3-​[[5,​7-​dihydroxy-​2,​2-​dimethyl-​6-​(2-​methyl-​1-​oxobutyl)​-​2H-​1-​benzopyran-​8-​yl]​methyl]​-​6-​ethyl-​4-​hydroxy-​5-​methyl-2H-​pyran-​2-​one [30] |
| 37.18 |  |  | 413.1598 [M-H]^-^ | C_23_H_26_O_7_ | TEDMF [43] |
| 37.51 |  |  | 499.1606 [M-H]^-^ | C_26_H_28_O_10_ | n.i. |
| 37.68 |  |  | 399.1441 [M-H]^-^ | C_22_H_24_O_7_ | italipyrone analog [22] |
| 37.78 |  |  | 465.2263 [M-H]^-^ | C_28_H_34_O_6_ | n.i. |
|  |  |  | 511.2326 [M+HCO_2_]^-^ |  |  |

^1^triethoxy-dimethoxy-flavone
