# Supplementary material for: Composition, Antioxidant Potential, and Antimicrobial Activity of Helichrysum plicatum DC. Various Extracts
Source: Plants (Basel). 2020 Mar 6;9(3):337. doi: 10.3390/plants9030337 (PMC7154845; doi:10.3390/plants9030337)
Supplement: Supplementary file 1 [file plants-09-00337-s001.zip › Table S1_EE tentative.docx]

**Table S1.** Tentative analysis of *Helichrysum plicatum* ethanol extract (EE).

| t_R_ (min) | | | UV λ_max_ (nm) | ToF-MS (*m/z*) | formula | compound |
| --- | --- | --- | --- | --- | --- | --- |
| ESI^+^ | ESI^-^ | LC-DAD |  | [ESI^+^/ESI^-^] |  |  |
|  | 1.99 |  |  | 179.0566 [M-H]^-^ | C_6_H_12_O_6_ | inositol [83] |
|  |  |  |  | 359.1197 [2M-H]^-^ |  |  |
| 2.02 | 2.02 |  |  | 161.0461 [M-H]- | C_6_H_10_O_5_ | n.i. |
|  |  |  |  | 207.0523 [M+HCO_2_]^-^ |  |  |
|  |  |  |  | 163.0606 [M+H]^+^ |  |  |
|  | 2.05 |  |  | 191.0565 [M-H]^-^ | C_7_H_12_O_6_ | quinic acid [91] |
|  |  |  |  | 237.0654 [M+HCO_2_]^-^ |  |  |
|  |  |  |  | 383.1214 [2M-H]^-^ |  |  |
| 2.22 |  |  |  | 273.1140 [M+H]^+^ | C_16_H_16_O_4_ | n.i. |
|  | 3.99 | 3.91 | 258; 288sh | 327.0734 [M-H]^-^ | C_14_H_16_O_9_ | 7-(*β*-D-glucopyranosyloxy)-5-hydroxy-1(3H)-isobenzofuranone [82] |
|  |  |  |  | 373.0795 [M+HCO_2_]^-^ |  |  |
|  |  |  |  | 655.1525 [2M-H]^-^ |  |  |
| 4.10 | 4.09 |  |  | 309.1202 [M+HCO_2_]^-^ | C_11_H_20_O_7_ | n.i. |
|  |  |  |  | 265.1272 [M+H]^+^ |  |  |
| 4.54 | 4.54 |  |  | 175.0614 [M-H]^-^ | C_7_H_12_O_5_ | n.i. |
|  |  |  |  | 177.0759 [M+H]^+^ |  |  |
| 4.81 | 4.81 |  |  | 175.0614 [M-H]^-^ | C_7_H_12_O_5_ | n.i. |
|  |  |  |  | 177.0754 [M+H]^+^ |  |  |
| 5.41 | 5.41 | 5.32 | 230; 256; 290 | 165.0197 [M-H]^-^ | C_8_H_6_O_4_ | 5,7-​dihydroxyphthalide [83] |
|  |  |  |  | 331.0476 [2M-H]^-^ |  |  |
|  |  |  |  | 167.0327 [M+H]^+^ |  |  |
|  | 5.92 | 5.83 | 228; 258; 288 | 387.0944 [M+HCO_2_]^-^ | C_15_H_18_O_9_ | 7-(*β*-D-glucopyranosyloxy)-5-methoxy-phthalide [83] |
| 6.12 | 6.11 | 6.01 | 245, 296sh; 326 | 353.0888 [M-H]^-^ | C_16_H_18_O_9_ | chlorogenic acid [86] |
|  |  |  |  | 399.0949 [M+HCO_2_]^-^ |  |  |
|  |  |  |  | 707.1832 [2M-H]^-^ |  |  |
|  |  |  |  | 355.1011 [M+H]^+^ |  |  |
| 7.17 | 7.19 | 7.08 | 240; 298sh; 324 | 179,0354 [M-H]^-^ | C_9_H_8_O_4_ | caffeic acid [87] |
|  |  |  |  | 359.0798 [2M-H]^-^ |  |  |
|  |  |  |  | 181.0473 [M+H]^+^ |  |  |
|  | 8.69 |  |  | 867.2348 [M-H]^-^ | C_42_H_44_O_20_ | arenariumoside V [53], arenariumoside VI [53], arenariumoside VII [53] |
|  |  |  |  | 913.2407 [M+HCO_2_]^-^ |  |  |
|  | 8.82 | 8.72 | 234; 288 | 481.1365 [M-H]^-^ | C_22_H_26_O_12_ | everlastoside M [88] |
|  |  |  |  | 527.1420 [M+HCO_2_]^-^ |  |  |
|  | 9.27 | 9.15 | 234; 260; 290 | 867.2338 [M-H]^-^ | C_42_H_44_O_20_ | tomoroside A [53] |
|  |  |  |  | 913.2402 [M+HCO_2_]^-^ |  |  |
| 9.37 |  | 9.26 | 234; 258; 286 | 181.0480 [M+H]^+^ | C_9_H_8_O_4_ | 5-​methoxy-​7-​hydroxyphthalide [84] |
| 9.44 | 9.46 | 9.35 | 234; 260; 282 | 433.1146 [M-H]^-^ | C_21_H_22_O_10_ | naringenin-5-*O*-glucoside [40] |
|  |  |  |  | 867.2333 [2M-H]^-^ |  |  |
|  |  |  |  | 435.1267 [M+H]^+^ |  |  |
|  | 9.66 |  |  | 197.0458 [M-H]^-^ | C_9_H_10_O_5_ | syringic acid [87] |
| 10.09 | 10.08 | 9.95 | 234; 282 | 433.1159 [M-H]^-^ | C_21_H_22_O_10_ | naringenin-4′-*O*-glucoside [48] |
|  |  |  |  | 479.1205 [M+HCO_2_]^-^ |  |  |
|  |  |  |  | 867.2348 [2M-H]^-^ |  |  |
|  |  |  |  | 913.2418 [2M+HCO_2_]^-^ |  |  |
|  |  |  |  | 435.1270 [M+H]^+^ |  |  |
| 11.32 | 11.32 | 11.16 | 256; 266sh; 304sh; 354 | 463.0894 [M-H]^-^ | C_21_H_20_O_12_ | quercetin-3-*O*-glucoside [4], hyperoside (quercetin-3-*O*-galactoside) [4] |
|  |  |  |  | 509.0955 [M+HCO_2_]^-^ |  |  |
|  |  |  |  | 465.1016 [M+H]^+^ |  |  |
|  | 13.00 |  |  | 515.1205 [M-H]^-^ | C_25_H_24_O_12_ | di-​*O*-​caffeoylquinic acid [4] |
|  |  |  |  | 1031.2455 [2M-H]^-^ |  |  |
|  | 13.56 | 13.42 |  | 433.1153 [M-H]^-^ | C_21_H_22_O_10_ | naringenin-​7-​*O*-glucoside [40] |
|  |  |  |  | 479,1211 [M+HCO_2_]^-^ |  |  |
| 14.56 | 14.54 | 14.39 | 234; 266; 304sh; 348 | 447.0946 [M-H]^-^ | C_21_H_20_O_11_ | kaempferol-​3-​*O*-​glucoside [50] |
|  |  |  |  | 493.1001 [M+HCO_2_]^-^ |  |  |
|  |  |  |  | 895.1929 [2M-H]^-^ |  |  |
|  |  |  |  | 449.1073 [M+H]^+^ |  |  |
| 15.16 | 15.16 | 14.97 | 234; 266; 336 | 431.0997 [M-H]^-^ | C_21_H_20_O_10_ | apigenin-7-*O*-glucoside [4] |
|  |  |  |  | 863.2038 [2M-H]^-^ |  |  |
|  |  |  |  | 433.1116 [M+H]^+^ |  |  |
| 15.52 | 15.47 |  |  | 515.1205 [M-H]^-^ | C_25_H_24_O_12_ | di-​*O*-​caffeoylquinic acid [4] |
|  |  |  |  | 1031.2412 [2M-H]^-^ |  |  |
|  |  |  |  | 517.1328 [M+H]^+^ |  |  |
| 15.88 | 15.86 | 15.67 | 268; 324 | 431.0994 [M-H]^-^ | C_21_H_20_O_10_ | apigenin-5-*O*-glucoside [40] |
|  |  |  |  | 477.1053 [M+HCO_2_]^-^ |  |  |
|  |  |  |  | 863.2038 [2M-H]^-^ |  |  |
|  |  |  |  | 433.1116 [M+H]^+^ |  |  |
| 17.71 | 17.73 | 17.52 | 236; 370 | 433.1157 [M-H] ^-^ | C_21_H_22_O_10_ | isosalipurposide [40] |
|  |  |  |  | 479.1205 [M+HCO_2_] ^-^ |  |  |
|  |  |  |  | 867.2341 [2M-H] ^-^ |  |  |
|  |  |  |  | 913.2398 [2M+HCO_2_] ^-^ |  |  |
|  |  |  |  | 435.1268 [M+H]^+^ |  |  |
| 19.79 | 19.76 | 19.55 | 232; 288 | 287.0571 [M-H] ^-^ | C_15_H_12_O_6_ | eriodictyol [49] |
|  |  |  |  | 333.0651 [M+HCO_2_] ^-^ |  |  |
|  |  |  |  | 575.1261 [2M-H] ^-^ |  |  |
|  |  |  |  | 289.0700 [M+H]^+^ |  |  |
| 20.49 | 20.45 | 20.23 | 266; 296sh; 326 | 609.1255 [M-H] ^-^ | C_30_H_26_O_14_ | helichrysoside [51] |
|  |  |  |  | 1219.2538 [2M-H]^-^ |  |  |
|  |  |  |  | 611.1389 [M+H]^+^ |  |  |
|  | 22.08 |  |  | 301.0363 [M-H]^-^ | C_15_H_10_O_7_ | quercetin [40] |
|  |  |  |  | 603.0804 [2M-H]^-^ |  |  |
| 22.35 | 22.32 | 22.09 | 254; 266; 302sh; 350 | 285.0415 [M-H]^-^ | C_15_H_10_O_6_ | luteolin [41] |
|  |  |  |  | 571.0896 [2M-H]^-^ |  |  |
|  |  |  |  | 287.0537 [M+H]^+^ |  |  |
| 23.07 | 23.09 | 22.83 | 234; 298; 310 | 639.3192 [M-H]^-^ | C_36_H_48_O_10_ | n.i. |
|  |  |  |  | 685.3256 [M+HCO_2_]^-^ |  |  |
|  |  |  |  | 1279.6430 [2M-H]^-^ |  |  |
|  |  |  |  | 641.3322 [M+H]^+^ |  |  |
| 23.41 | 23.38 | 23.13 | 234; 266; 300sh; 314 | 593.1310 [M-H]^-^ | C_30_H_26_O_13_ | tiliroside [52] |
|  |  |  |  | 639.1371 [M+HCO_2_]^-^ |  |  |
|  |  |  |  | 1187.2657 [2M-H]^-^ |  |  |
|  |  |  |  | 1233.2698 [2M+HCO_2_]^-^ |  |  |
|  |  |  |  | 595.1432 [M+H]^+^ |  |  |
| 23.91 | 23.89 | 23.67 | 232; 266; 314 | 593.1309 [M-H]^-^ | C_30_H_26_O_13_ | tiliroside analog [52] |
|  |  |  |  | 639.1391 [M+HCO_2_]^-^ |  |  |
|  |  |  |  | 595.1435 [M+H]^+^ |  |  |
| 24.45 | 24.44 | 24.20 | 234; 290 | 271.0623 [M-H]^-^ | C_15_H_12_O_5_ | naringenin [40] |
|  |  |  |  | 317.0682 [M+HCO_2_]^-^ |  |  |
|  |  |  |  | 273.0749 [M+H]^+^ |  |  |
| 27.90 |  |  |  | 323.1481 [M+H]^+^ | C_17_H_22_O_6_ | acylphloroglucinol derivative [47] |
| 25.65 | 25.65 | 25.35 | 232; 268; 302sh; 338 | 269.0462 [M-H]^-^ | C_15_H_10_O_5_ | apigenin [40] |
|  |  |  |  | 315.0555 [M+HCO_2_]^-^ |  |  |
|  |  |  |  | 271.0593 [M+H]^+^ |  |  |
| 25.76 | 25.71 |  |  | 309.0991 [M-H]^-^ | C_15_H_18_O_7_ | 7-(2,3-dihydroxy-3-methylbutoxy)-5-hydroxy-6-methoxy-2H-1-benzopyran-2-one [32] |
|  |  |  |  | 665.2108 [2M+HCO_2_]^-^ |  |  |
|  |  |  |  | 311.1111 [M+H]^+^ |  |  |
| 25.94 | 25.93 | 25.65 | 232; 268; 296sh; 316; 364 | 285.0413 [M-H]^-^ | C_15_H_10_O_6_ | kaempferol [41] |
|  |  |  |  | 331.0474 [M+HCO_2_]^-^ |  |  |
|  |  |  |  | 287.0536 [M+H]^+^ |  |  |
| 26.39 | 26.38 | 26.09 | 268; 296sh; 312 | 635.1411 [M-H]^-^ | C_32_H_28_O_14_ | n.i. |
|  |  |  |  | 681.1500 [M+HCO_2_]^-^ |  |  |
|  |  |  |  | 1271.2856 [2M-H]^-^ |  |  |
|  |  |  |  | 637.1536 [M+H]^+^ |  |  |
|  | 26.53 |  |  | 329.2344 [M-H]^-^ | C_18_H_34_O_5_ | pinellic acid [96], tianshic acid [97] |
| 26.63 | 26.67 | 26.31 | 234; 298; 308 | 785.3549 [M-H]^-^ | C_38_H_58_O_17_ | n.i. |
|  |  |  |  | 831.3616 [M+HCO_2_]^-^ |  |  |
|  |  |  |  | 787.3692 [M+H]^+^ |  |  |
| 27.57 | 27.57 | 27.25 | 236; 288; 338 | 293.1038 [M-H]^-^ | C_15_H_18_O_6_ | helipyrone C [24] |
|  |  |  |  | 295.1164 [M+H]^+^ |  |  |
|  | 28.29 |  |  | 351.1619 [M-H]^-^ | C_22_H_24_O_4_ | [3,​4-​dihydro-​5,​7-​dihydroxy-​2-​(4-​methyl-​3-​penten-​1-​yl)​-​2H-​1-​benzopyran-​6-​yl]​phenyl-methanone [81] |
| 28.60 | 28.60 |  |  | 585.1620 [M-H]^-^ | C_22_H_34_O_18_ | n.i. |
|  |  |  |  | 587.1746 [M+H]^+^ |  |  |
| 29.32 | 29.31 | 28.98 | 230; 290 | 235.0983 [M-H]^-^ | C_13_H_16_O_4_ | 3-prenyl-2,4,6-trihydroxyacetophenone [30], 4,​6-​dimethoxy-​5-​(2-​methyl-​1-​propen-​1-​yl)​-1,​3-​benzodioxole [80] |
|  |  |  |  | 517.2107 [2M+HCO_2_]^-^ |  |  |
|  |  |  |  | 237.1102 [M+H]^+^ |  |  |
|  | 29.44 |  |  | 417.1567 [M-H]^-^ | C_22_H_26_O_8_ | plicatipyrone analog [26] |
| 29.57 | 29.57 | 29.12 | 230; 276; 322sh; 372 | 599.1777 [M-H]^-^ | C_30_H_32_O_13_ | n.i. |
|  |  |  |  | 601.1893 [M+H]^+^ |  |  |
| 29.87 | 29.86 |  |  | 355.1200 [M-H]^-^ | C_20_H_20_O_6_ | helinivene A [104], piperitol [105], 1-​[6-​(acetyloxy)​-​2,​3,​4-​trimethoxyphenyl]​-​3-​phenyl- 2-​propen-​1-​one [106] |
|  |  |  |  | 357.1336 [M+H]^+^ |  |  |
| 30.81 | 30.81 | 30.41 | 230; 290 | 417.1596 [M-H]^-^ | C_22_H_26_O_8_ | plicatipyrone [26] |
|  |  |  |  | 419.1678 [M+H]^+^ |  |  |
| 31.17 | 31.17 |  |  | 313.2398 [M-H]^-^ | C_18_H_34_O_4_ | n.i. |
|  |  |  |  | 315.2505 [M+H]^+^ |  |  |
| 31.33 | 31.31 |  |  | 297.1146 [M-H]^-^ | C_18_H_18_O_4_ | phenyl[2,​4,​6-​trihydroxy-​3-​(3-​methyl-​2-​buten-​1-​yl)​phenyl]​-methanone [47], [2,​6-​dihydroxy-​4-​[(3-​methyl-​2-​buten-​1-​yl)​oxy]​phenyl]​phenyl-methanone [47] |
|  |  |  |  | 595.2387 [2M-H]^-^ |  |  |
|  |  |  |  | 299.1254 [M+H]^+^ |  |  |
| 31.48 | 31.48 |  |  | 461.1835 [M-H]^-^ | C_24_H_30_O_9_ | n.i. |
|  |  |  |  | 463.1939 [M+H]^+^ |  |  |
| 31.71 | 31.68 |  |  | 465.2293 [M-H]^-^ | C_28_H_34_O_6_ | n.i. |
|  |  |  |  | 467.2324 [M+H]^+^ |  |  |
| 31.74 | 31.74 | 31.38 | 228; 292 | 387.1465 [M-H]^-^ | C_21_H_24_O_7_ | arenol [27] |
|  |  |  |  | 389.1513 [M+H]^+^ |  |  |
| 32.01 |  |  |  | 433.1755 [M+H]^+^ | C_30_H_24_O_3_ | n.i. |
| 32.13 |  | 31.77 | 228; 278; 324; 376 | 345.0890 [M+H]^+^ | C_25_H_12_O_2_ | n.i. |
| 32.16 | 32.16 |  |  | 373.1671 [M-H]^-^ | C_21_H_26_O_6_ | 4-​(3-​methoxy-​3-​oxo-​1-​propen-​1-​yl)​-​2-​(3-​methyl-​2-​buten-​1-​yl)​phenyl-3-​(acetyloxy)​-​butanoic acid ester [90] |
|  |  |  |  | 375.1720 [M+H]^+^ |  |  |
|  | 32.20 |  |  | 293.2121 [M-H]^-^ | C_18_H_30_O_3_ | PUFA^1^ [99–101] |
|  |  |  |  | 339.2191 [M+HCO_2_]^-^ |  |  |
| 32.26 | 32.26 |  |  | 417.1572 [M-H]^-^ | C_22_H_26_O_8_ | plicatipyrone analog [26] |
|  |  |  |  | 835.3167 [2M-H]^-^ |  |  |
|  |  |  |  | 419.1606 [M+H]^+^ |  |  |
| 32.33 | 32.32 | 31.99 | 222; 234sh; 278; 284 | 361.1672 [M-H]^-^ | C_20_H_26_O_6_ | n.i. |
|  |  |  |  | 363.1719 [M+H]^+^ |  |  |
| 32.36 | 32.35 |  |  | 357.1358 [M-H]^-^ | C_20_H_22_O_6_ | pinoresinol [102] |
|  |  |  |  | 359.1416 [M+H]^+^ |  |  |
|  | 32.51 |  |  | 319.1566 [M-H]^-^ | C_18_H_24_O_5_ | n.i. |
| 32.71 | 32.71 | 32.35 | 234; 294 | 401.1624 [M-H]^-^ | C_22_H_26_O_7_ | arzanol [27] |
|  |  |  |  | 403.1649 [M+H]^+^ |  |  |
|  |  |  |  | 805.3273 [2M+H]^+^ |  |  |
| 32.87 | 32.87 |  |  | 445.1888 [M-H]^-^ | C_24_H_30_O_8_ | heliarzanol [23] |
|  |  |  |  | 447.1879 [M+H]^+^ |  |  |
| 33.15 | 33.14 |  |  | 401.1622 [M-H]^-^ | C_22_H_26_O_7_ | cycloarzanol [23] |
|  |  |  |  | 849.3323 [2M+HCO_2_]^-^ |  |  |
|  |  |  |  | 403.1655 [M+H]^+^ |  |  |
| 33.38 | 33.37 | 33.01 | 204; 216 | 449.1617 [M-H]^-^ | C_26_H_26_O_7_ | n.i. |
|  |  |  |  | 451.1630 [M+H]^+^ |  |  |
|  | 33.38 |  |  | 303.1614 [M-H]^-^ | C_18_H_24_O_4_ | 2,​3-​dihydro-​5,​7-​dihydroxy-​3-​methyl-​8-​(3-​methyl-​2-​butenyl)​-​2-​(1-​methylethyl)​-4H-​1-benzopyran-​4-​one [39] |
| 33.38 |  |  |  | 413.2051 [M+H]^+^ | C_28_H_28_O3 | n.i. |
|  | 33.41 |  |  | 371.1519 [M-H]^-^ | C_21_H_24_O_6_ | 7-​acetyl-​5'-​ethyl-​4,​6-​dihydroxy-​4'-​methyl-​5-​(3-​methyl-​2-​buten-​1-​yl)​-spiro[benzofuran-​2(3H)​,​2'(3'H)​-​furan]​-​3'-​one [24] |
| 33.61 | 33.60 |  |  | 403.1419 [M-H]^-^ | C_21_H_24_O_8_ | helipyrone diacetate [25] |
|  |  |  |  | 405.1439 [M+H]^+^ |  |  |
|  | 33.80 |  |  | 415.1783 [M-H]^-^ | C_23_H_28_O_7_ | methylarzanol [23] |
|  | 34.00 |  |  | 415.1784 [M-H]^-^ | C_23_H_28_O_7_ | 3-​[1-​[3-​acetyl-​2,​4,​6-​trihydroxy-​5-​(3-​methyl-​2-​buten-​1-​yl)​phenyl]​ethyl]​-​6-​ethyl-​4-​hydroxy-​5-​methyl-2H-pyran-​2-​one [23] |
| 34.16 | 34.15 | 33.76 | 294 | 463.1776 [M-H]^-^ | C_23_H_28_O_10_ | 1-​[2,​6-​bis(acetyloxy)​-​4-​[[4-​(acetyloxy)​-​3-​[(acetyloxy)​methyl]​-​2-​buten-​1-​yl]​oxy]​phenyl]​-1-​butanone [47] |
|  |  |  |  | 465.1780 [M+H]^+^ |  |  |
| 34.42 | 34.43 | 34.05 | 278 | 293.2136 [M-H]^-^ | C_18_H_30_O_3_ | PUFA [99–101] |
|  |  |  |  | 295.2182 [M+H]^+^ |  |  |
| 34.46 | 34.46 |  |  | 429.1940 [M-H]^-^ | C_24_H_30_O_7_ | 4-​hydroxy-​5,​6-​dimethyl-​3-​[[2,​4,​6-​trihydroxy-​3-​(3-​methyl-​2-​buten-​1-​yl)​-​5-​(2-​methyl-​1-​oxobutyl)​phenyl]​methyl]​-2H-​pyran-​2-​one [24] |
|  |  |  |  | 431.1938 [M+H]^+^ |  |  |
|  | 34.65 | 34.26 | 290 | 455.2094 [M-H]^-^ | C_26_H_32_O_7_ | 3-​[[3-​acetyl-​5-​(3,​7-​dimethyl-​2,​6-​octadien-​1-​yl)​-​2,​4,​6-​trihydroxyphenyl]​methyl]​-​4-​hydroxy-​5,​6-​dimethyl-2H-​pyran-​2-​one [29] |
|  | 34.65 | 34.26 | 290 | 417.1580 [M-H]^-^ | C_22_H_26_O_8_ | plicatipyrone analog [26] |
|  | 34.70 |  |  | 429.1941 [M-H]^-^ | C_24_H_30_O_7_ | 6-​ethyl-​4-​hydroxy-​5-​methyl-​3-​[[2,​4,​6-​trihydroxy-​3-​(3-​methyl-​2-​buten-​1-​yl)​-​5-​(2-​methyl-​1-​oxopropyl)​phenyl]​methyl]​-2H-​pyran-​2-​one [23] |
|  | 34.92 |  |  | 587.1947 [M-H]^-^ | C_33_H_32_O_10_ | n.i. |
| 34.92 |  |  |  | 589.1895 [M+H]^+^ | C_29_H_32_O_13_ | n.i. |
| 35.12 |  |  |  | 445.2081 [M+H]^+^ | C_21_H_32_O_10_ | n.i. |
|  | 35.28 | 34.89 | 234; 294 | 469.2263 [M-H]^-^ | C_20_H_38_O_12_ | n.i. |
| 35.29 |  |  |  | 471.2232 [M+H]^+^ | C_23_H_34_O_10_ | n.i. |
| 35.47 |  |  |  | 593.2582 [M+H]^+^ | C_30_H_40_O_12_ | n.i. |
| 35.49 |  |  |  | 577.2334 [M+H]^+^ | C_40_H_32_O_4_ | n.i. |
| 35.71 |  |  |  | 451.2356 [M+H]^+^ | C_24_H_34_O_8_ | n.i. |
| 35.80 | 35.78 |  |  | 457.2265 [M-H]^-^ | C_26_H_34_O_7_ | 3-​[1-​[3-​acetyl-​2,​4,​6-​trihydroxy-​5-​(3-​methyl-​2-​buten-​1-​yl)​phenyl]​heptyl]​-​4-​hydroxy-​6-​methyl-2H-​pyran-​2-​one [23], 3-​[[2,​4-​dihydroxy-​6-​methoxy-​5-​(3-​methyl-​2-​buten-​1-​yl)​-​3-​(2-​methyl-​1-​oxobutyl)​phenyl]​methyl]​-​6-​ethyl-​4-​hydroxy-​5-​methyl-2H-​pyran-​2-​one [30], 3-​[[2,​4-​dihydroxy-​6-​methoxy-​3-​(3-​methyl-​2-​buten-​1-​yl)​-​5-​(2-​methyl-​1-​oxobutyl)​phenyl]​methyl]​-​6-​ethyl-​4-​hydroxy-​5-​methyl-2H-​pyran-​2-​one [22] |
|  |  |  |  | 459.2276 [M+H]^+^ |  |  |
| 35.95 | 35.95 |  |  | 433.2411 [M-H]^-^ | C_21_H_38_O_9_ | ainsliaside E [69] |
|  |  |  |  | 435.2427 [M+H]^+^ |  |  |
| 36.07 |  |  |  | 447.2233 [M+H]^+^ | C_32_H_30_O_2_ | n.i. |
|  | 36.12 |  |  | 483.2421 [M-H]^-^ | C_28_H_36_O_7_ | 3-​[[3-​acetyl-​5-​(3,​7-​dimethyl-​2,​6-​octadienyl)​-​2,​4,​6-​trihydroxyphenyl]​methyl]​-​4-​hydroxy-​5-​methyl-​6-​propyl-2H-​pyran-​2-​one [29], 3-​[[3-​(​3,​7-​dimethyl-​2,​6-​octadien-​1-​yl)​-​2,​4,​6-​trihydroxy-​5-​(2-​methyl-​1-​oxopropyl)​phenyl]​methyl]​-​4-​hydroxy-​5,​6-​dimethyl-2H-​pyran-​2-​one [24] |
|  | 36.30 |  |  | 429.1763 [M-H]^-^ | C_20_H_30_O_10_ | n.i. |
|  |  |  |  | 475.1800 [M+HCO_2_]^-^ |  |  |
| 36.31 |  |  |  | 477.1798 [M+H]^+^ | C_21_H_32_O_12_ | n.i. |
|  | 36.46 |  |  | 497.2584 [M-H]^-^ | C_29_H_38_O_7_ | 3-​[[3,​7-​dimethyl-​2,​6-​octadien-​1-​yl]​-​2,​4,​6-​trihydroxy-​5-​(2-​methyl-​1-​oxobutyl)​phenyl]​methyl]​-​4-​hydroxy-​5,​6-​dimethyl-2H-pyran-​2-​one [24] |
|  | 36.48 |  |  | 449.2371 [M-H]^-^ | C_21_H_38_O_10_ | n.i. |
|  | 36.60 |  |  | 591.2658 [M-H]^-^ | C_27_H_44_O_14_ | n.i. |
| 36.63 |  |  |  | 593.2634 [M+H]^+^ | C_23_H_44_O_17_ | n.i. |
| 36.65 |  |  |  | 413.2606 [M+H]^+^ | C_41_H_36_O_4_ | n.i. |
|  | 38.86 |  |  | 489.1971 [M-H]^-^ | C_22_H_34_O_12_ | n.i. |
| 38.86 |  |  |  | 491.1960 [M+H]^+^ | C_18_H_34_O_15_ | n.i. |
| 37.01 |  |  |  | 621.2952 [M+H]^+^ | C_25_H_48_O_17_ | n.i. |
|  | 37.08 |  |  | 485.2218 [M-H]^-^ | C_20_H_38_O_3_ | gymnospermin [74] |
|  | 37.21 |  |  | 461.2721 [M-H]^-^ | C_30_H_38_O_4_ | helilupolone [54] |
| 37.34 | 37.34 |  |  | 413.2708 [M-H]^-^ | C_26_H_38_O_4_ | ent-kaurane derivative [73] |
|  |  |  |  | 415.2776 [M+H]^+^ |  |  |
|  | 37.48 |  |  | 501.3018 [M-H]^-^ | C_33_H_42_O_4_ | n.i. |
|  | 37.69 |  |  | 443.2812 [M-H]^-^ | C_27_H_40_O_5_ | n.i. |
|  | 37.77 |  |  | 427.2864 [M-H]^-^ | C_27_H_40_O_4_ | n.i. |

^1^polyunsaturated fatty acid
