# Supplementary material for: Composition, Antioxidant Potential, and Antimicrobial Activity of Helichrysum plicatum DC. Various Extracts
Source: Plants (Basel). 2020 Mar 6;9(3):337. doi: 10.3390/plants9030337 (PMC7154845; doi:10.3390/plants9030337)
Supplement: Supplementary file 1 [file plants-09-00337-s001.zip › Table S2_DCME tentative.docx]

**Table S2.** Tentative analysis of *Helichrysum plicatum* dichloromethane extract (DCME).

| t_R_ (min) | | | UV λ_max_ (nm) | ToF-MS (*m/z*) | formula | compound |
| --- | --- | --- | --- | --- | --- | --- |
| ESI^+^ | ESI^-^ | LC-DAD |  | [ESI^+^/ESI^-^] |  |  |
| 5.16 |  |  |  | 201.0751 [M+H]^+^ | C_9_H_12_O_5_ | 1,6-dihydroxy-4-oxo-2-cyclohexene-1-acetic acid methyl ester [92] |
| 5.41 | 5.44 | 5.34 | 256; 292 | 167.0339 [M+H]^+^ | C_8_H_6_O_4_ | 5,7-​dihydroxyphthalide [83] |
|  |  |  |  | 165.0196 [M-H]^-^ |  |  |
|  |  |  |  | 331.0473 [2M-H]^-^ |  |  |
| 5.71 |  |  |  | 187.0958 [M+H]^+^ | C_9_H_14_O_4_ | tetrahydrojacarone [93] |
| 5.87 |  |  |  | 119.0701 [M+H]^+^ | C_5_H_10_O_3_ | n.i. |
| 9.43 | 9.43 | 9.26 | 228; 258; 292 | 181.0492 [M+H]^+^ | C_9_H_8_O_4_ | 5-​methoxy-​7-​hydroxyphthalide [84] |
|  |  |  |  | 179.0350 [M-H]^-^ |  |  |
| 10.24 |  |  |  | 297.1325 [M+H]^+^ | C_15_H_20_O_6_ | 4-​[3,​5-​dihydroxy-​4-​(1-​oxobutyl)​phenoxy]​-​2-​methyl-butanoic acid [47], 4-​[3,​5-​dihydroxy-​4-​(2-​methyl-​1-​oxopropyl)​phenoxy]​-​2-​methyl-butanoic acid [47], 1-​[2,​6-​dihydroxy-​4-​[[4-​hydroxy-​3-​(hydroxymethyl)​-​2-​buten-​1-​yl]​oxy]​phenyl]​-1-butanone [47] |
| 10.36 |  |  |  | 193.0488 [M+H]^+^ | C_10_H_8_O_4_ | 3',4'-methylenedioxycinnamic acid [89] |
| 11.01 |  |  |  | 197.1164 [M+H]^+^ | C_11_H_16_O_3_ | loliolide [94] |
| 12.29 |  |  |  | 227.0908 [M+H]^+^ | C_11_H_14_O_5_ | n.i. |
| 16.84 | 16.83 |  |  | 311.1117 [M+H]^+^ | C_15_H_18_O_7_ | n.i. |
|  |  |  |  | 309.0990 [M-H]^-^ |  |  |
| 17.46 | 17.45 | 17.25 | 234; 296 | 311.1120 [M+H]^+^ | C_15_H_18_O_7_ | n.i. |
|  |  |  |  | 309.0990 [M-H]^-^ |  |  |
|  | 19.33 |  |  | 367.1560 [M-H]^-^ | C_22_H_24_O_5_ | n.i. |
| 20.01 | 20.00 |  |  | 311.1117 [M+H]^+^ | C_15_H_18_O_7_ | n.i. |
|  |  |  |  | 309.0990 [M-H]^-^ |  |  |
| 20.59 |  |  |  | 309.1303 [M+H]_+_ | C_16_H_20_O_6_ | 4,​5-​dimethoxy-​6-​(2-​methyl-​1-​propen-​1-​yl)​-1,​3-​benzenediol-1,​3-​diacetate [79] |
| 21.53 |  |  |  | 255.1217 [M+H]^+^ | C_13_H_18_O_5_ | 1-​(4,​6-​dihydroxy-​2,​3-​dimethoxyphenyl)​-​2-​methyl-1-​butanone [43,79,80], 1-​(2,​4-​diethoxy-​6-​hydroxy-​3-​methoxyphenyl)​-ethanone [43,79,80], 4,​6-​dimethoxy-​α-​(1-​methylethyl)​-1,​3-​benzodioxole-​5-​methanol [43,79,80] |
| 21.77 |  |  |  | 309.1232 [M+H]^+^ | C_16_H_20_O_6_ | 4-​[3,​5-​dihydroxy-​4-​(2-​methyl-​1-​oxopropyl)​phenoxy]​-​2-​methyl-​2-​butenoic acid methyl ester [47] |
| 22.40 | 22.39 | 22.13 | 284 | 393.1535 [M+H]^+^ | C_20_H_24_O_8_ | 2-​[4-​(​1,​3-​dihydroxypropyl)-​2-​methoxyphenoxy]​-​3-​hydroxy-1-​(4-​hydroxy-​3-​methoxyphenyl)-​1-propanone [95] |
|  |  |  |  | 391.1409 [M-H]^-^ |  |  |
| 24.43 | 24.42 | 24.17 | 234; 288 | 273.0751 [M+H]^+^ | C_15_H_12_O_5_ | naringenin [40] |
|  |  |  |  | 271.0620 [M-H]^-^ |  |  |
|  |  |  |  | 317.0685 [M+HCO_2_]^-^ |  |  |
| 24.71 | 24.72 |  |  | 325.1275 [M+H]^+^ | C_16_H_20_O_7_ | 2',3'-​dihydroxypuberulin [31] |
|  |  |  |  | 323.1145 [M-H]^-^ |  |  |
| 24.90 |  |  |  | 323.1480 [M+H]^+^ | C_17_H_22_O_6_ | acylphloroglucinol derivative [47] |
| 24.95 | 24.94 |  |  | 373.1268 [M+H]^+^ | C_20_H_20_O_7_ | 3,5,6,7,8-​pentamethoxyflavone [42] |
|  |  |  |  | 371.1148 [M-H]^-^ |  |  |
| 25.22 | 25.22 | 24.95 | 252; 316 | 373.1268 [M+H]^+^ | C_20_H_20_O_7_ | 3',4',5,6,7-pentamethoxyflavone [43] |
|  |  |  |  | 371.1149 [M-H]^-^ |  |  |
| 25.73 | 25.73 | 25.42 | 196; 204; 208; 216; 220; 236; 290; 238sh | 311.1114 [M+H]^+^ | C_15_H_18_O_7_ | 7-(2,3-dihydroxy-3-methylbutoxy)-5-hydroxy-6-methoxy-2H-1-benzopyran-2-one [32] |
|  |  |  |  | 309.0994 [M-H]^-^ |  |  |
|  |  |  |  | 619.2070 [2M-H]^-^ |  |  |
|  | 26.40 |  |  | 393.1563 [M-H]^-^ | C_20_H_26_O_8_ | sesquiterpene derivative [55–57] |
|  | 26.54 |  |  | 329.2341 [M-H]^-^ | C_18_H_34_O_5_ | pinellic acid [96], tianshic acid [97] |
|  | 26.60 |  |  | 313.0726 [M-H]^-^ | C_17_H_14_O_6_ | dihydroxy-dimethoxyflavone [44] |
| 26.74 | 26.75 |  |  | 395.1696 [M+H]^+^ | C_20_H_26_O_8_ | sesquiterpene derivative [55–57] |
|  |  |  |  | 393.1566 [M-H]^-^ |  |  |
|  | 26.90 | 26.59 | 192; 198; 206; 212; 220; 248sh; 272 | 291.0883 [M-H]^-^ | C_15_H_16_O_6_ | coumarin derivative [33,34] |
| 27.00 |  |  |  | 455.1690 [M+H]^+^ | C_25_H_26_O_8_ | n.i. |
| 27.30 | 27.31 |  |  | 353.1585 [M+H]^+^ | C_18_H_24_O_7_ | n.i. |
|  |  |  |  | 351.1461 [M-H]^-^ |  |  |
| 27.57 | 27.56 |  |  | 295.1161 [M+H]^+^ | C_15_H_18_O_6_ | helipyrone C [24] |
|  |  |  |  | 293.1044 [M-H]^-^ |  |  |
|  | 27.91 |  |  | 263.1299 [M-H]^-^ | C_15_H_20_O_4_ | 2-​methyl-​1-​[2,​4,​6-​trihydroxy-​3-​(3-​methyl-​2-​buten-​1-​yl)​phenyl]​-1-propanone [47], 1-​[2,​6-​dihydroxy-​4-​[(3-​methyl-​2-​buten-​1-​yl)​oxy]​phenyl]​-​2-​methyl-1-propanone [47], 1-​[2,​6-​dihydroxy-​4-​[(3-​methyl-​2-​buten-​1-​yl)​oxy]​phenyl]​-1-butanone [47], 1-​[2,​4,​6-​trihydroxy-​3-​(3-​methyl-​2-​buten-​1-​yl)​phenyl]​-1-butanone [47], 1-​(3,​4-​dihydro-​5,​7-​dihydroxy-​2,​2-​dimethyl-​2H-​1-​benzopyran-​6-​yl)​-​2-​methyl-1-propanone [30] |
| 28.24 | 28.23 |  |  | 353.1729 [M+H]^+^ | C_22_H_24_O_4_ | [3,​4-​dihydro-​5,​7-​dihydroxy-​2-​(4-​methyl-​3-​penten-​1-​yl)​-​2H-​1-​benzopyran-​6-​yl]​phenyl- methanone [81] |
|  |  |  |  | 351.1619 [M-H]^-^ |  |  |
| 28.33 | 28.32 |  |  | 373.1281 [M+H]^+^ | C_20_H_20_O_7_ | (3,4,5,6,7-pentamethoxy-2-benzofuranyl)phenylmethanone [98] |
|  |  |  |  | 371.1152 [M-H]^-^ |  |  |
| 28.47 | 28.46 |  |  | 325.1243 [M+H]^+^ | C_16_H_20_O_7_ | 7-(2,3-dihydroxy-3-methylbutoxy)-5,6-dimethoxy-2H-1-benzopyran-2-one [35] |
|  |  |  |  | 323.1147 [M-H]^-^ |  |  |
|  | 28.55 |  |  | 291.0879 [M-H]^-^ | C_15_H_16_O_6_ | bisnorhelipyrone [25] |
| 28.67 | 28.67 |  |  | 391.1731 [M+H]^+^ | C_21_H_26_O_7_ | n.i. |
|  |  |  |  | 389.1620 [M-H]^-^ |  |  |
| 29.49 | 29.52 | 29.12 | 276; 298sh; 328sh; 370 | 331.0762 [M+H]^+^ | C_24_H_10_O_2_ + C_21_H_24_O_8_ | n.i. |
|  |  |  |  | 405.1470 [M+H]^+^ |  |  |
|  |  |  |  | 403.1412 [M-H]^-^ |  |  |
| 29.80 | 29.78 | 29.52 | 232; 292 | 309.1270 [M+H]^+^ | C_16_H_20_O_6_ | helipyrone B [24] |
|  |  |  |  | 307.1197 [M-H]^-^ |  |  |
|  |  |  |  | 353.1268 [M+HCO_2_]^-^ |  |  |
|  | 30.34 | 30.00 | 238sh; 284 | 373.1307 [M-H]^-^ | C_20_H_22_O_7_ | 3-​[[3-​acetyl-​2,​4,​6-​trihydroxy-​5-​(3-​methyl-​2-​buten-​1-​yl)​phenyl]​methyl]​-​4-​hydroxy-​6-​methyl-2H-​pyran-​2-​one [23] |
|  | 30.52 |  |  | 397.1311 [M-H]^-^ | C_22_H_22_O_7_ | 5,7,4'-trihydroxy-3,6-dimethoxy-3'-prenylflavone [46] |
| 30.76 | 30.76 | 30.42 | 230; 288 | 419.1610 [M+H]^+^ | C_22_H_26_O_8_ | plicatipyrone [26] |
|  |  |  |  | 417.1575 [M-H]^-^ |  |  |
|  | 31.22 | 30.86 | 242; 284 | 275.0929 [M-H]^-^ | C_15_H_16_O_5_ | 1-[2-[1-[(acetyloxy)methyl]ethenyl]-2,3-dihydro-3-hydroxy-5-benzofuranyl]ethanone [51] |
| 31.73 | 31.75 |  |  | 389.1508 [M+H]^+^ | C_21_H_24_O_7_ | arenol [27] |
|  |  |  |  | 387.1463 [M-H]^-^ |  |  |
|  | 31.82 |  |  | 381.1361 [M-H]^-^ | C_22_H_22_O_6_ | 3-​(acetyloxy)​-​2,​3-​dihydro-​5,​7-​dihydroxy-​6-​(3-​methyl-​2-​butenyl)​-​2-​phenyl-4H-​1-​benzopyran-​4-​one [38] |
| 32.12 |  | 31.76 | 242; 278; 326; 376 | 345.0882 [M+H]^+^ | C_25_H_12_O_2_ | n.i. |
| 32.72 | 32.73 |  |  | 403.1646 [M+H]^+^ | C_22_H_26_O_7_ | arzanol [27] |
|  |  |  |  | 401.1627 [M-H]- |  |  |
| 33.38 | 33.38 | 33.01 | 232; 286 | 373.1537 [M+H]^+^ | C_21_H_24_O_6_ | 7-​acetyl-​5'-​ethyl-​4,​6-​dihydroxy-​4'-​methyl-​5-​(3-​methyl-​2-​buten-​1-​yl)​-spiro[benzofuran-​2(3H)​,​2'(3'H)​-​furan]​-​3'-​one [24] |
|  |  |  |  | 371.1520 [M-H]^-^ |  |  |
|  |  |  |  | 743.3058 [2M-H]^-^ |  |  |
| 33.61 | 33.60 |  |  | 405.1407 [M+H]^+^ | C_21_H_24_O_8_ | helipyrone diacetate [25] |
|  |  |  |  | 403.1422 [M-H]^-^ |  |  |
| 33.83 |  | 33.42 |  | 295.2168 [M+H]^+^ | C_18_H_30_O_3_ | PUFA^1^ [99–101] |
| 34.11 |  | 33.76 |  | 295.2173 [M+H]^+^ | C_18_H_30_O_3_ | PUFA [99–101] |
|  |  |  |  | 589.4214 [2M+H]^+^ |  |  |
| 34.16 | 34.16 |  |  | 465.1776 [M+H]^+^ | C_23_H_28_O_10_ | 1-​[2,​6-​bis(acetyloxy)​-​4-​[[4-​(acetyloxy)​-​3-​[(acetyloxy)​methyl]​-​2-​buten-​1-​yl]​oxy]​phenyl]​-1-​butanone [47] |
|  |  |  |  | 463.1795 [M-H]^-^ |  |  |
| 34.21 |  |  |  | 507.1848 [M+H]^+^ | C_25_H_30_O_11_ | n.i. |
| 34.38 |  |  |  | 279.1519 [M+H]^+^ | C_18_H_30_O_2_ | dihydro-​5-​(5,​8-​tetradecadienyl)​-2(3H)​-​furanone [47] |
| 34.43 |  | 34.05 | 278 | 295.2188 [M+H]^+^ | C_18_H_30_O_3_ | PUFA [99–101] |
| 34.65 | 34.64 | 34.26 | 248sh; 292 | 419.1561 [M+H]^+^ | C_22_H_26_O_8_ | plicatipyrone analog [26] |
|  |  |  |  | 417.1583 [M-H]^-^ |  |  |
| 35.11 |  |  |  | 417.2481 [M+H]^+^ | C_21_H_36_O_8_ | eudesmane derivative [65] |
| 35.22 | 35.21 |  |  | 389.2175 [M+H]^+^ | C_19_H_32_O_8_ | n.i. |
|  |  |  |  | 387.2186 [M-H]^-^ |  |  |
| 35.42 | 35.41 |  |  | 373.2250 [M+H]^+^ | C_23_H_32_O_4_ | decahydro-​4-​hydroxy-​8a-​methyl-​5-​methylene-​3-​(1-​methylethyl)​-​1-​naphthalenyl ester-4-​methoxy-benzoic acid [107] |
|  |  |  |  | 371.2240 [M-H]^-^ |  |  |
| 35.71 | 35.70 | 35.31 | 250sh; 282; 360 | 451.2309 [M+H]^+^ | C_24_H_34_O_8_ | n.i. |
|  |  |  |  | 449.2350 [M-H]^-^ |  |  |
| 35.94 |  |  |  | 431.2629 [M+H]^+^ | C_22_H_38_O_8_ | n.i. |
| 35.96 | 35.95 | 35.55 | 248; 360 | 435.2373 [M+H]^+^ | C_24_H_34_O_7_ | n.i. |
|  |  |  |  | 433.2390 [M-H]^-^ |  |  |
|  | 36.20 |  |  | 445.1882 [M-H]^-^ | C_24_H_30_O_8_ | n.i. |
|  | 36.43 |  |  | 445.1880 [M-H]^-^ | C_24_H_30_O_8_ | n.i. |
|  | 36.46 |  |  | 471.2044 [M-H]^-^ | C_26_H_32_O_8_ | n.i. |
| 36.47 |  |  |  | 433.2233 [M+H]^+^ | C_24_H_32_O_7_ | n.i. |
| 36.62 |  |  |  | 445.2819 [M+H]^+^ | C_23_H_40_O_8_ | n.i. |
| 36.71 | 36.70 |  |  | 417.2520 [M+H]^+^ | C_25_H_36_O_5_ | *ent*-​kaurane derivative [73], eudesmane derivative [65] |
|  |  |  |  | 415.2499 [M-H]^-^ |  |  |
| 36.84 | 36.84 |  |  | 401.2595 [M+H]^+^ | C_25_H_36_O_4_ | *ent*-​kaurane derivative [73] |
|  |  |  |  | 399.2546 [M-H]^-^ |  |  |
| 36.87 | 36.86 |  |  | 491.1958 [M+H]^+^ | C_29_H_30_O_7_ | 6-[(6-ethyl-4-hydroxy-5-methyl-2-oxo-2H-pyran-3-yl)methyl]-2,3-dihydro-5,7-dihydroxy-8-(3-methyl-2-buten-1-yl)-2-phenyl-4H-1-benzopyran-4-one [47] |
|  |  |  |  | 489.1927 [M-H]^-^ |  |  |
|  |  |  |  | 535.2013 [M+HCO_2_]^-^ |  |  |
|  | 36.89 |  |  | 459.2033 [M-H]^-^ | C_25_H_32_O_8_ | athrolide C [71] |
|  | 37.07 |  |  | 485.2196 [M-H]^-^ | C_27_H_34_O_8_ | 2-​methyl-​1-​[2,​4,​6-​trihydroxy-​5-​[(1S)​-​1-​(4-​hydroxy-​6-​methoxy-​1,​3-​benzodioxol-​5-​yl)​-​2-​methylpropyl]​-​3-​(3-​methyl-​2-​butenyl)​phenyl]​-1-propanone [30] |
| 37.14 |  |  |  | 263.2330 [M+H]^+^ | C_16_H_32_O | hexadecanal [103] |
| 37.21 |  |  |  | 357.2370 [M+H]^+^ | C_21_H_34_O_3_ | diterpenoid derivative [75–77] |
| 37.22 | 37.22 |  |  | 431.2726 [M+H]^+^ | C_26_H_38_O_5_ | *ent*-kaurane derivative [73] |
|  |  |  |  | 429.2663 [M-H]^-^ |  |  |
| 37.22 |  |  |  | 459.3040 [M+H]^+^ | C_26_H_44_O_5_ | n.i. |
| 37.33 |  |  |  | 413.2633 [M+H]^+^ | C_24_H_38_O_4_ | 5-​(acetyloxy)​-​α-​ethenyldecahydro-​α,​3a,​5,​7b-​tetramethyl-1H-​cyclopropa[a]​naphthalene-​4-​propanol acetate [78] |
|  | 37.36 |  |  | 413.2713 [M-H]^-^ | C_26_H_38_O_4_ | *ent*-kaurane derivative [73] |
|  | 37.49 |  |  | 473.2201 [M-H]^-^ | C_26_H_34_O_8_ | athrolide D [71] |
|  | 37.70 |  |  | 443.2821 [M-H]^-^ | C_27_H_40_O_5_ | n.i. |

^1^polyunsaturated fatty acid
